# Supplementary material for: A novel rapalog shows improved safety vs. efficacy in a human organoid model of polycystic kidney disease
Source: Stem Cell Reports. 2025 Jan 23;20(2):102395. doi: 10.1016/j.stemcr.2024.102395 (PMC11864154; doi:10.1016/j.stemcr.2024.102395)
Supplement: Document S1. Figures S1–S7 [file mmc1.pdf]

Stem Cell Reports, Volume *20*

Supplemental Information

A novel rapalog shows improved safety vs. efficacy in a human organoid model of polycystic kidney disease

Ramila E. Gulieva, Parvaneh Ahmadvand, and Benjamin S. Freedman

SUPPLEMENTAL FIGURE S1

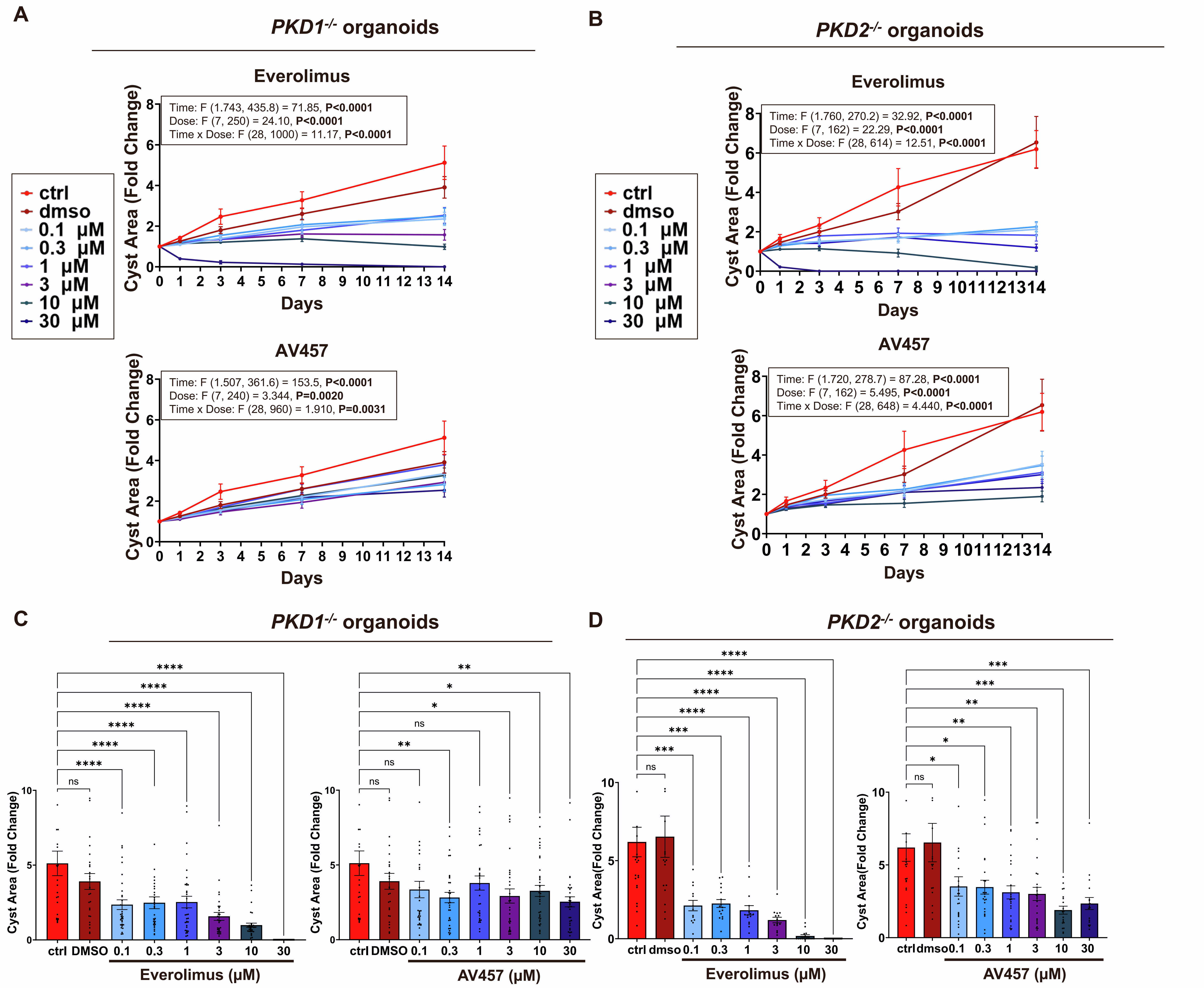

Supplemental Figure S1. mTOR inhibitors reduce cyst growth in *$PKD1^{-/-}$* and *$PKD2^{-/-}$* organoids. (A) Cyst area fold change measurements for organoids treated with AV457 or everolimus (mean ± s.e.m.; n > 40 organoids per condition, pooled from 5 independent experiments using *$PKD1^{-/-}$* organoids. Boxes show two-way ANOVA analysis for Time and Dose dependence). (B) Cyst area fold change measurements from 3 independent experiments using *$PKD2^{-/-}$* organoids. (C) Detailed analysis of cyst area fold change on day 14 for *$PKD1^{-/-}$*, with statistical analysis, and (D) corresponding analysis for *$PKD2^{-/-}$* organoids. Statistical significance is indicated as follows: ns, not significant; *p ≤ 0.05; **p ≤ 0.01; ***p ≤ 0.001; ****p ≤ 0.0001.

SUPPLEMENTAL FIGURE S2

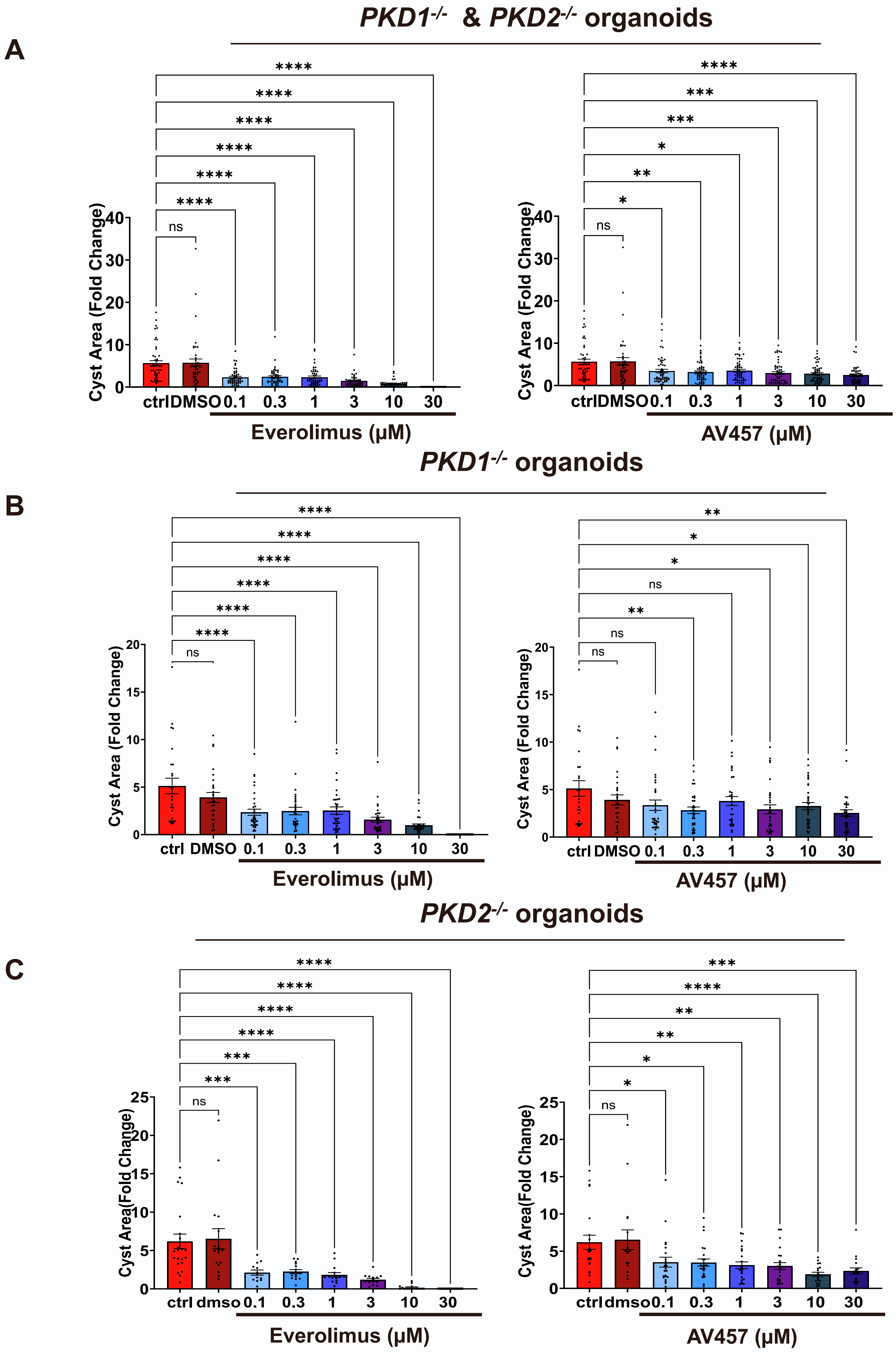

Supplementary Figure S2. mTOR inhibitors reduce PKD cyst growth over time. (A) Full-range data for both *PKD1*$^{-/-}$ and *PKD2*$^{-/-}$ organoids from Figure 2D. (B) Full-range data for *PKD1*$^{-/-}$ organoids from Supplementary Figure S1C and (C) for *PKD2*$^{-/-}$ organoids from Supplementary Figure S1D. Statistical significance is indicated as follows: ns, not significant; *p ≤ 0.05; **p ≤ 0.01; ***p ≤ 0.001; ****p ≤ 0.0001.

SUPPLEMENTAL FIGURE S3

A

PKD1$^{-/-}$ & *PKD2*$^{-/-}$ organoids

Everolimus

Tukey's multiple comparisons test	Mean Diff.	Summary	Adjusted P Value
0.1 vs. 0.3	-0.1139	ns	>0.9999
0.1 vs. 1	-0.02167	ns	>0.9999
0.1 vs. 3	0.8368	ns	0.8372
0.1 vs. 10	1.556	ns	0.1145
**0.1 vs. 30**	**2.293**	**	**0.0011**
0.3 vs. 1	0.0922	ns	>0.9999
0.3 vs. 3	0.9507	ns	0.727
0.3 vs. 10	1.669	ns	0.0679
**0.3 vs. 30**	**2.407**	***	**0.0005**
1 vs. 3	0.8585	ns	0.8145
1 vs. 10	1.577	ns	0.1004
**1 vs. 30**	**2.315**	***	**0.0009**
3 vs. 10	0.7188	ns	0.918
3 vs. 30	1.456	ns	0.1668
10 vs. 30	0.7375	ns	0.8845

AV457

Tukey's multiple comparisons test	Mean Diff.	Summary	Adjusted P Value
0.1 vs. 0.3	0.2561	ns	0.9999
0.1 vs. 1	-0.08259	ns	>0.9999
0.1 vs. 3	0.4638	ns	0.9948
0.1 vs. 10	0.6424	ns	0.9659
0.1 vs. 30	0.9539	ns	0.7855
0.3 vs. 1	-0.3387	ns	0.9993
0.3 vs. 3	0.2078	ns	>0.9999
0.3 vs. 10	0.3863	ns	0.9984
0.3 vs. 30	0.6978	ns	0.9497
1 vs. 3	0.5464	ns	0.9861
1 vs. 10	0.725	ns	0.9353
1 vs. 30	1.036	ns	0.7049
3 vs. 10	0.1785	ns	>0.9999
3 vs. 30	0.49	ns	0.9935
10 vs. 30	0.3115	ns	0.9997

B

PKD1$^{-/-}$ organoids

Everolimus

Tukey's multiple comparisons test	Mean Diff.	Summary	Adjusted P Value
0.1 vs. 0.3	-0.1271	ns	>0.9999
0.1 vs. 1	-0.176	ns	>0.9999
0.1 vs. 3	0.7831	ns	0.824
0.1 vs. 10	1.37	ns	0.1504
**0.1 vs. 30**	**2.356**	***	**0.0001**
0.3 vs. 1	-0.04892	ns	>0.9999
0.3 vs. 3	0.9101	ns	0.7072
0.3 vs. 10	1.497	ns	0.0973
**0.3 vs. 30**	**2.483**	****	**<0.0001**
1 vs. 3	0.9591	ns	0.631
1 vs. 10	1.546	ns	0.068
**1 vs. 30**	**2.532**	****	**<0.0001**
3 vs. 10	0.5869	ns	0.958
3 vs. 30	1.573	ns	0.0552
10 vs. 30	0.9863	ns	0.5229

AV457

Tukey's multiple comparisons test	Mean Diff.	Summary	Adjusted P Value
0.1 vs. 0.3	0.5346	ns	0.9936
0.1 vs. 1	-0.4351	ns	0.9982
0.1 vs. 3	0.4311	ns	0.9984
0.1 vs. 10	0.08955	ns	>0.9999
0.1 vs. 30	0.8178	ns	0.9271
0.3 vs. 1	-0.9697	ns	0.843
0.3 vs. 3	-0.1035	ns	>0.9999
0.3 vs. 10	-0.445	ns	0.9977
0.3 vs. 30	0.2833	ns	0.9999
1 vs. 3	0.8662	ns	0.9103
1 vs. 10	0.5247	ns	0.9935
1 vs. 30	1.253	ns	0.5794
3 vs. 10	-0.3416	ns	0.9996
3 vs. 30	0.3867	ns	0.9992
10 vs. 30	0.7283	ns	0.9551

C

PKD2$^{-/-}$ organoids

Everolimus

Tukey's multiple comparisons test	Mean Diff.	Summary	Adjusted P Value
0.1 vs. 0.3	-0.1313	ns	>0.9999
0.1 vs. 1	0.3012	ns	>0.9999
0.1 vs. 3	0.9255	ns	0.9867
0.1 vs. 10	1.949	ns	0.5538
0.1 vs. 30	2.123	ns	0.3059
0.3 vs. 1	0.4325	ns	0.9998
0.3 vs. 3	1.057	ns	0.9626
0.3 vs. 10	2.08	ns	0.3968
0.3 vs. 30	2.254	ns	0.1656
1 vs. 3	0.6243	ns	0.9985
1 vs. 10	1.648	ns	0.7086
1 vs. 30	1.822	ns	0.4467
3 vs. 10	1.024	ns	0.9711
3 vs. 30	1.197	ns	0.888
10 vs. 30	0.1735	ns	>0.9999

AV457

Tukey's multiple comparisons test	Mean Diff.	Summary	Adjusted P Value
0.1 vs. 0.3	0.04958	ns	>0.9999
0.1 vs. 1	0.4079	ns	0.9998
0.1 vs. 3	0.513	ns	0.9991
0.1 vs. 10	1.63	ns	0.6799
0.1 vs. 30	1.173	ns	0.927
0.3 vs. 1	0.3583	ns	>0.9999
0.3 vs. 3	0.4634	ns	0.9995
0.3 vs. 10	1.58	ns	0.7032
0.3 vs. 30	1.123	ns	0.9385
1 vs. 3	0.1051	ns	>0.9999
1 vs. 10	1.222	ns	0.9039
1 vs. 30	0.7649	ns	0.9934
3 vs. 10	1.117	ns	0.9355
3 vs. 30	0.6598	ns	0.9972
10 vs. 30	-0.4568	ns	0.9998

Supplementary Figure S3. Statistical summary for cyst fold change in *PKD1*$^{-/-}$ and *PKD2*$^{-/-}$ organoids.
(A) One-way ANOVA followed by Tukey's multiple comparisons test was conducted for Figure 2D and (B-C) Supplementary Figure S1C-D. Statistical significance is indicated as follows: ns, not significant; *p ≤ 0.05; **p ≤ 0.01; ***p ≤ 0.001; ****p ≤ 0.0001.

SUPPLEMENTAL FIGURE S4

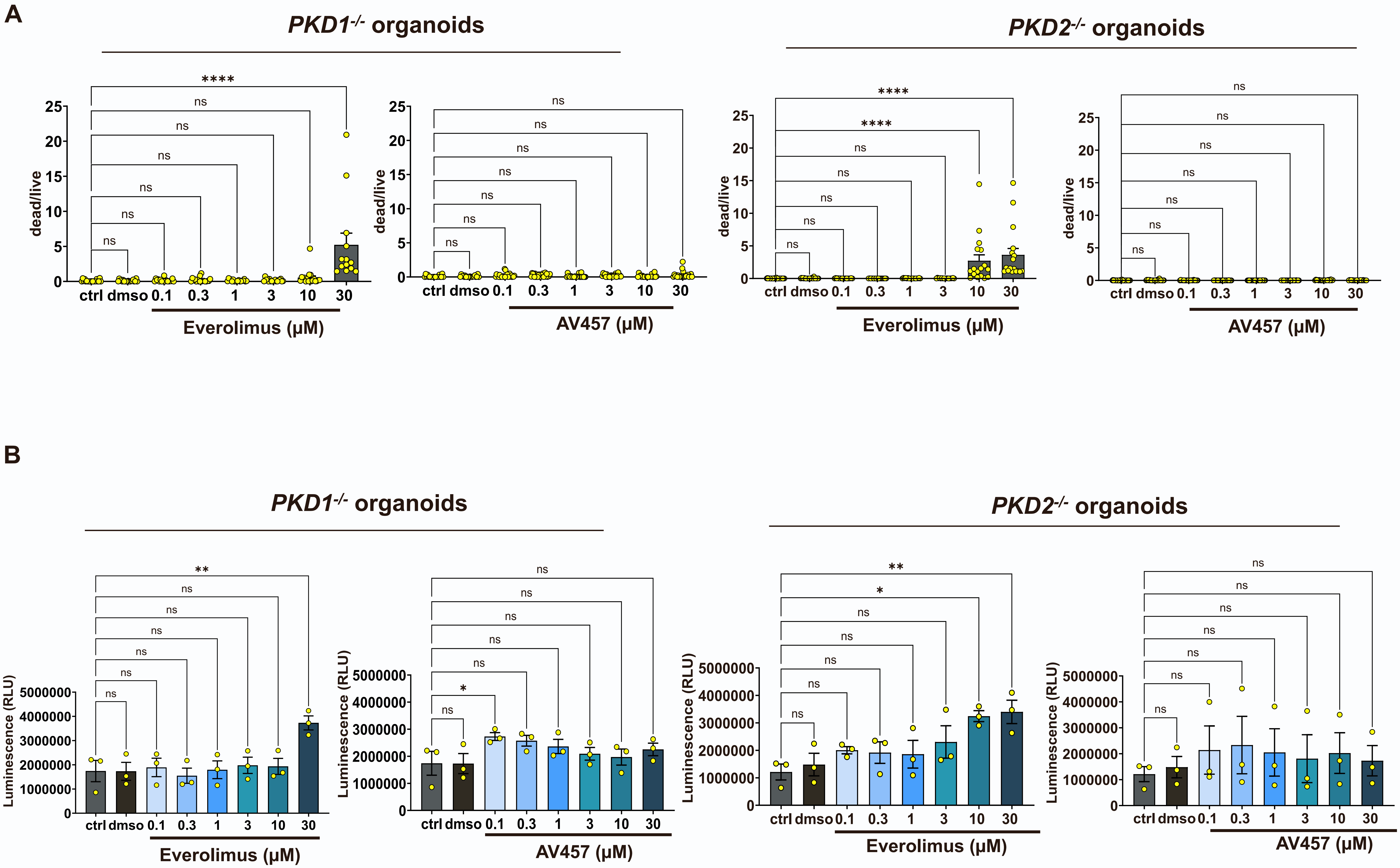

Supplemental Figure S4. AV457 shows reduced toxicity relative to everolimus in *PKD1*$^{-/-}$ and *PKD2*$^{-/-}$ cysts. (A) Live/Dead analysis using thresholding to measure the area (mean ± s.e.m.; n ≥ 20 organoids per condition pooled from 4 experiments with *PKD1*$^{-/-}$ or 3 experiments with *PKD2*$^{-/-}$ organoids). (B) LDH assays on day 7 of treatment (mean ± s.e.m.; n = 3 independent experiments). ns, not significant; *, p ≤ 0.05; **, p ≤ 0.01; ***, p ≤ 0.001; ****, p ≤ 0.0001.

SUPPLEMENTAL FIGURE S5

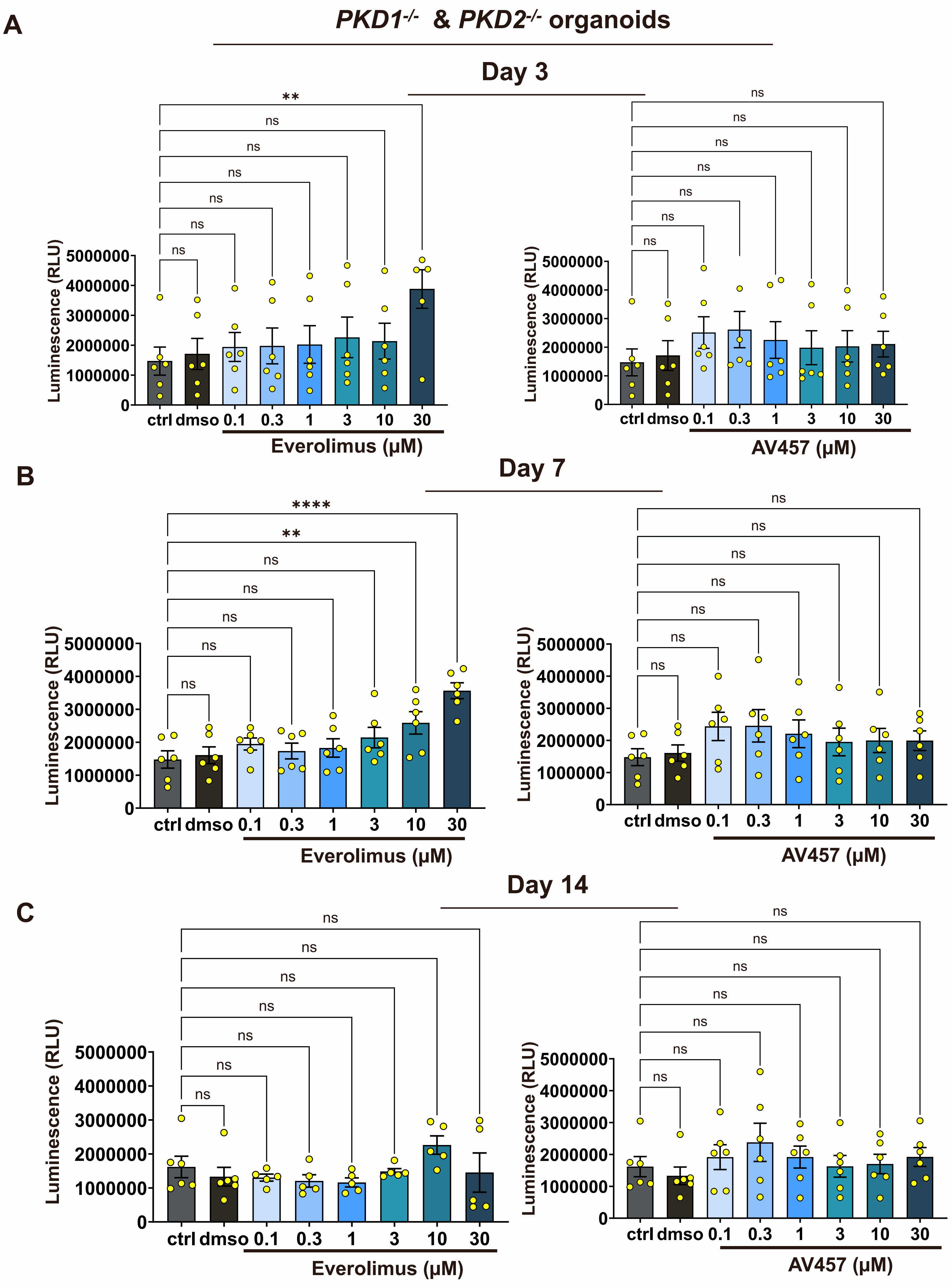

Supplemental Figure S5. LDH activity in PKD cysts is time sensitive. (A) LDH activity measured on day 3, (B) day 7, and (C) day 14 (mean ± s.e.m.; n = 6 independent experiments, three with *PKD1*$^{-/-}$ and three with *PKD2*$^{-/-}$ organoids; ns, not significant; *, $p \leq 0.05$; **, $p \leq 0.01$; ***, $p \leq 0.001$; ****, $p \leq 0.0001$).

SUPPLEMENTAL FIGURE S6

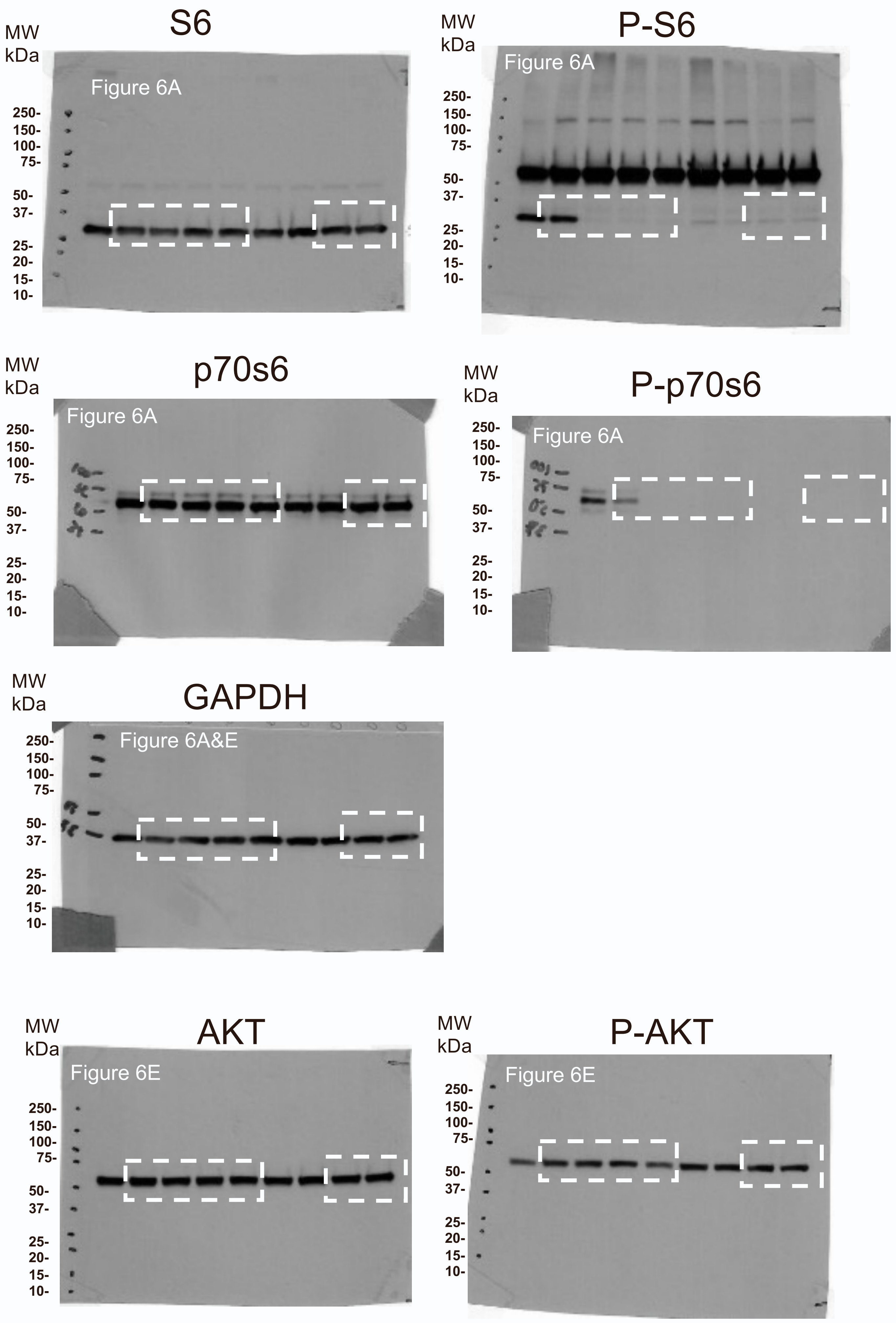

Supplementary Figure S6. mTOR inhibitors show target engagement in kidney organoids. Full representative Western blots from Figure 6. Cropped sections are boxed in a white outline.

SUPPLEMENTAL FIGURE S7

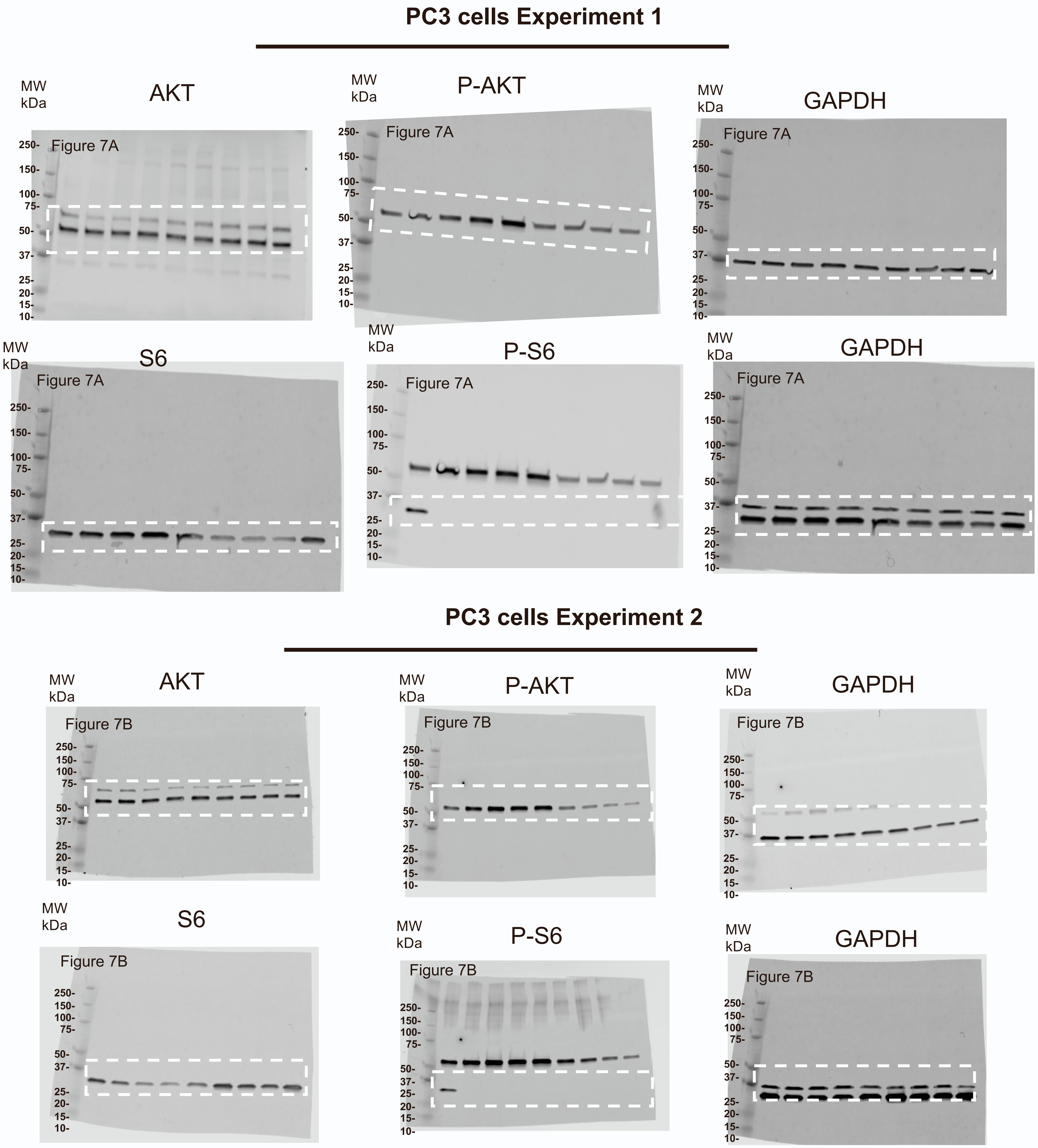

Supplementary Figure S7. **mTOR inhibitors show target engagement in PC3 cells.** Full representative Western blots from Figure 7. Cropped sections used in Figure 7 are outlined in white. For Experiment 1, the blots for S6, AKT, and their corresponding GAPDH initially had the control sample at the end of the blot. For consistency, these were cropped and repositioned with the control sample at the beginning, while the other blots remained unchanged.
