# Supplementary material for: A novel rapalog shows improved safety vs. efficacy in a human organoid model of polycystic kidney disease
Source: Stem Cell Reports. 2025 Jan 23;20(2):102395. doi: 10.1016/j.stemcr.2024.102395 (PMC11864154; doi:10.1016/j.stemcr.2024.102395)
Supplement: Document S2. Article plus supplemental information [file mmc2.pdf]

Stem Cell Reports
Article
OPEN ACCESS

A novel rapalog shows improved safety vs. efficacy in a human organoid model of polycystic kidney disease

Ramila E. Gulieva,[1] Parvaneh Ahmadvand,[2] and Benjamin S. Freedman[1,2,3,*]
[1]Department of Medicine, Division of Nephrology, Institute for Stem Cell & Regenerative Medicine, and Kidney Research Institute, University of Washington School of Medicine, Seattle, WA 98109, USA
[2]Plurexa LLC, Seattle, WA 98109, USA
[3]Lead contact
*Correspondence: benof@uw.edu
https://doi.org/10.1016/j.stemcr.2024.102395

SUMMARY

The mammalian target of rapamycin (mTOR) pathway is a therapeutic target in polycystic kidney disease (PKD), but mTOR inhibitors such as everolimus have failed to show efficacy at tolerated doses in clinical trials. Here, we introduce AV457, a novel rapalog developed to reduce side effects, and assess its dose-dependent safety and efficacy versus everolimus in $PKD1^{-/-}$ and $PKD2^{-/-}$ human kidney organoids, which form cysts in a PKD-specific way. Both AV457 and everolimus reduce cyst growth over time. At intermediate doses, AV457 exhibits an improved safety profile relative to everolimus, with comparable efficacy. Target engagement assays confirm mTOR pathway inhibition and greater selectivity of AV457 for mTOR complex 1 versus complex 2, compared to everolimus. AV457 thus provides a more favorable balance of safety and efficacy for PKD compared to everolimus and merits further consideration as an investigational therapeutic.

INTRODUCTION

Polycystic kidney disease (PKD) is the leading monogenic cause of kidney failure, marked by expansion of tiny tubules into macroscopic, fluid-filled sacs. PKD is commonly caused by loss of *PKD1* or *PKD2*, encoding polycystin-1 or polycystin-2. There remains a substantial need for PKD therapeutics, as the only available drug, tolvaptan, has limited efficacy and can cause side effects that preclude use in many patients (Torres et al., 2018). Activation of mTOR (mammalian or mechanistic target of rapamycin) is associated with PKD, and mTOR inhibitors such as everolimus and sirolimus showed promise in preclinical animal models, suggesting a therapeutic approach (Shillingford et al., 2006; Wahl et al., 2006; Wu et al., 2009). In human clinical trials, however, everolimus (5 mg/day) reduced cystic kidney growth but failed to adequately protect kidney function, while sirolimus (2 mg/day) showed no beneficial effect, compared to placebo (Serra et al., 2010; Walz et al., 2010).

In these trials, the doses used were intentionally limited to achieve tolerability for mTOR inhibitors, which are associated with side effects of increased risk of diabetes and immunosuppression (Lamming et al., 2012; Trelinska et al., 2015; Arriola Apelo, Neuman et al., 2016; Yao et al., 2016; Schreiber et al., 2019). However, the dose required to inhibit mTOR activity is organ specific and may need to be higher to achieve efficacy for PKD (Canaud et al., 2010; Jardine et al., 2013). The mTOR pathway includes two complexes, mTORC1 and mTORC2. While sirolimus and everolimus have a higher affinity for mTORC1, they can also inhibit mTORC2 (Lamming et al., 2012; Arriola Apelo et al., 2016). mTORC2 plays a specific role as an effector of insulin signaling to regulate glucose metabolism and may be responsible for many of the side effects of mTOR inhibitors (Sarbassov et al., 2004). To avoid such side effects, mTORC1-selective inhibitors have recently been developed, which exhibit greater specificity, although these have not yet been tested for PKD (Schreiber et al., 2019).

As animals did not accurately predict mTOR responses in human PKD, a human model capable of dose-dependent assays would be valuable for testing mTOR inhibitors. Primary human kidney cells can form spheroid structures in three-dimensional cultures, but this phenomenon occurs even with non-PKD cells and is therefore not specific for the disease (Neufeld et al., 1992; Xu et al., 2022). Recently, human kidney organoids have been derived from induced pluripotent stem (iPS) cells, which form cysts in a PKD-specific manner (Freedman et al., 2015; Cruz et al., 2017; Li et al., 2022; Tran et al., 2022). This provides a species-specific and higher-throughput platform to analyze therapeutics, compared to animal models (Czerniecki et al., 2018; Tran et al., 2022).

Previously, we have shown that the gene signatures of mTOR signaling are increased in PKD organoids, consistent with a potential role for mTOR in organoid cystogenesis (Cruz et al., 2017). A preliminary screen of chemicals suggested possible efficacy of rapamycin, although results were inconsistent among doses and genotypes (Tran et al., 2022). Thus mTOR inhibitors have not been tested in kidney organoids in detail. Here, we apply the human PKD organoid system to conduct preclinical safety and efficacy assessment of a novel rapalog, not previously tested in

© 2025 The Authors.
Published by Elsevier Inc. on behalf of International Society for Stem Cell Research.
This is an open access article under the CC BY license (http://creativecommons.org/licenses/by/4.0/).

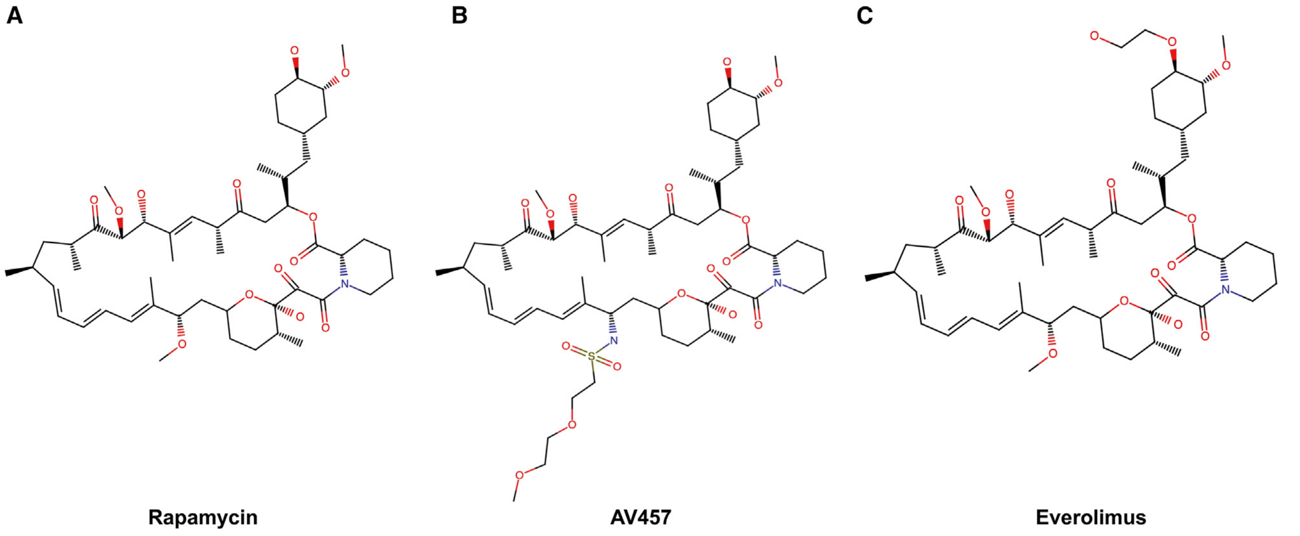

Figure 1. AV457 is a novel rapalog

Structures of (A) rapamycin, (B) AV457, and (C) everolimus. The International Union of Pure and Applied Chemistry name for AV457 is 1R,9S,12S,15R,18R,19R,21R,23S,30S,32S,35R,16E,24E,26E,28E)-12-{(R)-2-[(1S,3R,4R)-4-(3-hydroxypropoxy)-3-methoxycyclohexyl]-1-methylethyl}-1,18-dihydroxy-19-methoxy-30-[2-(2-methoxyethoxy)ethylsulfonylamino]-15,17,21,23,29,35-hexamethyl-11,36-dioxa-4-azatricyclo[30.3.1.0^{4,9}]hexatriaconta-16,24,26,28-tetraene-2,3,10,14,20-pentone.

any PKD model system, to gain insight into the therapeutic potential of this compound, compared to everolimus.

RESULTS

mTOR inhibitors reduce cyst growth in PKD organoids

A novel rapalog was synthesized by substituting a (2-methoxyethoxy)ethylsulfonylamino group for a methoxy group at carbon 16 of rapamycin. The resultant compound, AV457, was distinct in chemical structure from both rapamycin and everolimus (Figures 1A–1C).

To evaluate the effect of AV457 on PKD cyst growth, we utilized our well-established model of human kidney organoids derived from human iPS cells with null mutations in *PKD1* or *PKD2*. Mature organoids (day 21 after plating) were transferred into suspension cultures for 10 days to form early cysts. Once organoids became cystic (day 30), individual cysts were transferred into 96-well plates and the treatment time course was initiated (Figure 2A).

AV457 was compared with everolimus, vehicle control (DMSO), and untreated conditions in side-by-side experiments. Each compound was added at doses of 0.1 μM, 0.3 μM, 1 μM, 3 μM, 10 μM, and 30 μM, and cyst area was quantified for each individual organoid over time on days 0, 1, 3, 7, and 14. Both AV457 and everolimus reduced cyst growth in a dose-dependent manner, which became more pronounced over time, relative to vehicle-treated or untreated control conditions (Figures 2B and 2C). By two-way analysis of variance (ANOVA), the effects of both compounds on cyst fold change were significantly influenced by both dose and time of treatment ($p < 0.0001$; Figure 2C). By day 14 of treatment, the endpoint of this study, the difference in cyst growth was approximately 2-fold for intermediate doses of mTOR inhibitors, compared to the controls, and reached a high level of statistical significance for each of the mTOR inhibitors at every dose (Figure 2D).

Based on dose-response plots of these data, the half-maximal inhibitory concentration (IC50) values for AV457 and everolimus were calculated to be 1.7–4.6 nM and 1.6–2.6 nM, respectively (Figures 3A–3C). This is similar to the reported IC50 of 1.6–2.4 nM for everolimus in a cell-free assay (Sedrani et al., 1998). A similar trend was observed in both *PKD1*$^{-/-}$ and *PKD2*$^{-/-}$ organoids (Figures S1A–S1D and S2A–S2C). Thus, the general strategy of mTOR inhibition demonstrated efficacy in reducing cyst growth in PKD organoids.

Everolimus exhibits secondary dose-dependent effects at high concentrations

Interestingly, at higher concentrations (3–30 μM), everolimus exhibited a prominent dose-dependent decline in cyst growth from the lower concentrations (0.1–1 μM), whereas AV457-treated organoids exhibited a more consistent effect throughout the dose range (Figures 2C and 2D). Comparing the individual concentrations, Tukey's multiple comparison test revealed a significant difference between the 30 μM treatment and concentrations ≤1 μM in everolimus-treated organoids but did not reveal

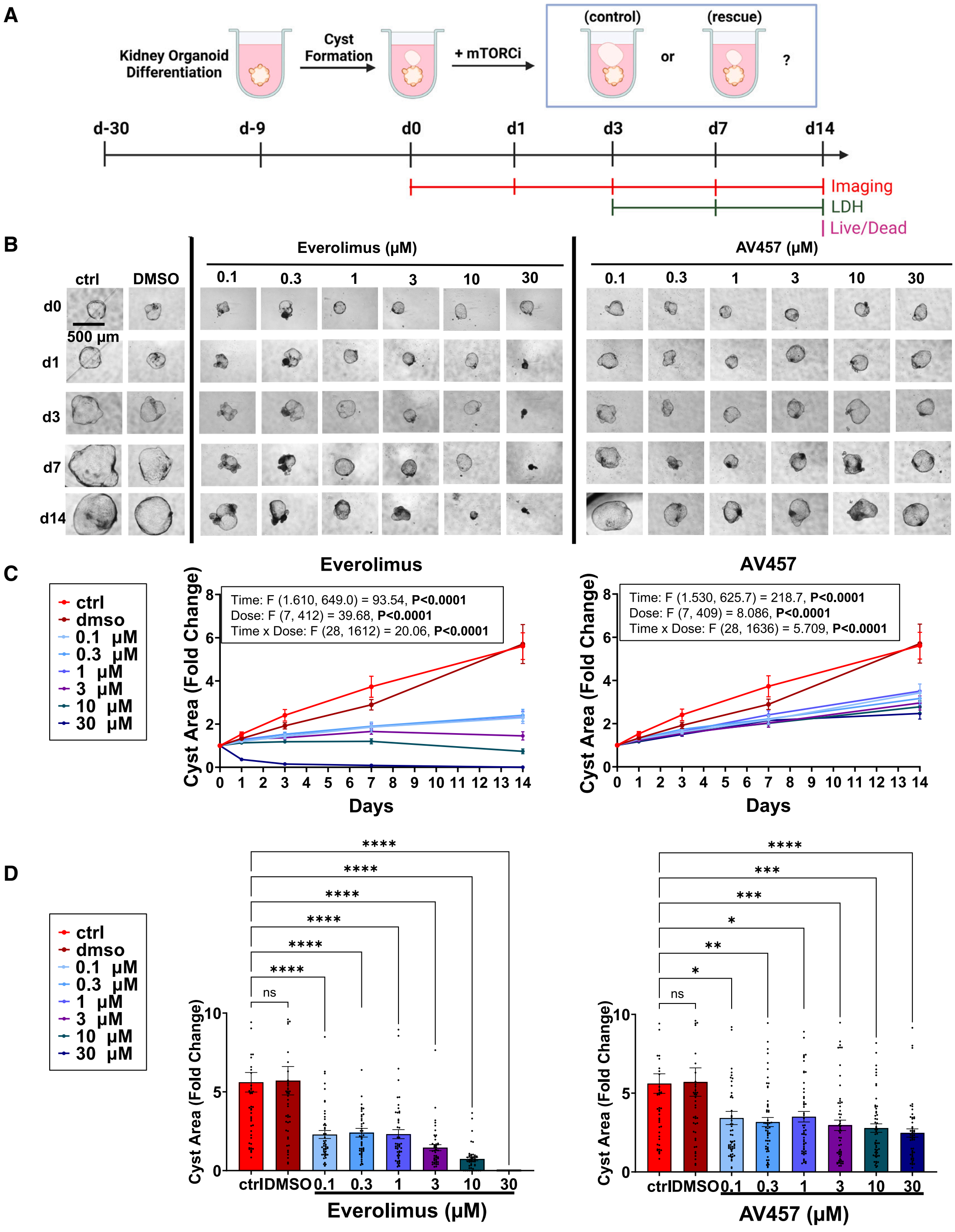

Figure 2. mTOR inhibitors reduce PKD cyst growth over time

(A) Schematic time course of the experiment.

(B and C) (B) Representative images of PKD cysts over time, with (C) quantification of cyst area fold change (mean ± SEM, $n > 40$ organoids per condition, pooled from 8 independent experiments, five with *PKD1*$^{-/-}$ and three with *PKD2*$^{-/-}$ organoids). Boxes show two-way ANOVA analysis for time and dose dependence.

(D) Bar charts of cyst area fold change on day 14 from the dataset shown in (C) with statistical analysis (ns, not significant; *, $p \leq 0.05$; **, $p \leq 0.01$; ***, $p \leq 0.001$; ****, $p \leq 0.0001$ by one-way ANOVA).

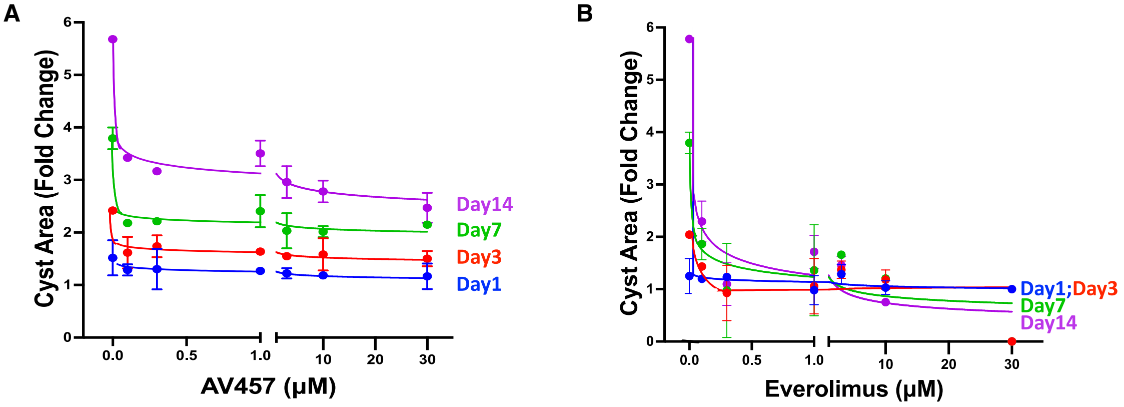

C

Compound	IC50 (nM)			
	Day1	Day3	Day7	Day14
**AV457 (0-30 μM)**	1.74 ± 0.09	2.23 ± 0.07	3.01 ± 0.15	4.68 ± 0.31
**Everolimus (0-30 μM)**	1.60 ± 0.13	1.73 ± 0.01	2.18 ± 0.49	2.65 ± 0.15

Figure 3. Dose-response curves of PKD organoid cyst growth in AV457 and everolimus treatment regimens

(A and B) (A) Plots of cyst area fold change in cysts treated with increasing concentrations of AV457 and (B) everolimus over a 14-day period. Graphs on right show extension of the x axis between 0 and 1 μM.

(C) IC50 values representing mean ± standard deviation (SD) of $n \geq 40$ organoids per condition pooled from 8 independent experiments, five with *PKD1*$^{-/-}$ and three with *PKD2*$^{-/-}$ organoids.

statistically significant differences between these doses in AV457-treated organoids (Figure S3A). This statistical result was preserved when *PKD1*$^{-/-}$ organoids were assessed alone but was not detectable when only *PKD2*$^{-/-}$ organoids were analyzed (Figures S3B and S3C).

We performed a more detailed comparative analysis of the relative effects of each mTOR inhibitor on day 14. In the lower half of the dose range (0.1–1 μM), everolimus had a slightly stronger effect than AV457, but this advantage appeared negligible at the dose of 1 μM and was not statistically significant at any dose (Figures 4A–4C). In the upper half of the dose range (3–30 μM), the effect of everolimus was more pronounced, compared to AV457, a finding that was statistically significant at each dose (Figures 4D–4F). Gross analysis of the cysts by phase-contrast microscopy further indicated degradation of the structures at the highest doses for everolimus (Figure 2B). AV457 and everolimus thus showed similar efficacies in reducing cyst growth at lower concentrations, but diverged at higher concentrations, with everolimus showing a secondary dose-dependent effect possibly due to cytotoxicity.

AV457 is less toxic than everolimus

As the preceding datasets suggested some toxicity at the higher doses in these experiments, and toxicity can masquerade as efficacy in studies of PKD cyst growth, we conducted a more rigorous safety analysis of these treatment conditions. Live-dead staining analysis at the 14-day endpoint detected statistically significant toxicity of everolimus at 10–30 μM, whereas AV457 did not exhibit significant toxicity even at the highest dose (Figures 5A and 5B). Similarly, lactate dehydrogenase (LDH) activity in the media of these organoid cultures on day 7 revealed toxicity of everolimus but not AV457 (Figure 5C). Trends for these live-dead and LDH analyses were generally similar for both *PKD1*$^{-/-}$ and *PKD2*$^{-/-}$ organoids, although the toxicity of everolimus at 10 μM was significant only in the *PKD2*$^{-/-}$ organoids (Figures S4A and S4B). LDH measurements were less sensitive earlier (day 3) or later (day 14), due to the cells being relatively healthy or already dead, respectively (Figures S5A–S5C). Thus, our experiments revealed overt toxicity of everolimus at 10 μM, whereas AV457 did not exhibit any measurable toxicity at doses up to 30 μM.

To test whether doses of everolimus below 10 μM might also exhibit detectable toxic effects, we applied a luminescence-based viability assay based on quantitation of adenosine triphosphate (CellTiter-Glo). Cisplatin, a known nephrotoxicant, was utilized as a positive control. In these experiments, treatment with 1 μM or 3 μM everolimus significantly reduced ATP levels, compared to the untreated control, while not reaching the dramatic cytotoxicity caused by cisplatin (Figure 5D). Overall, these experiments

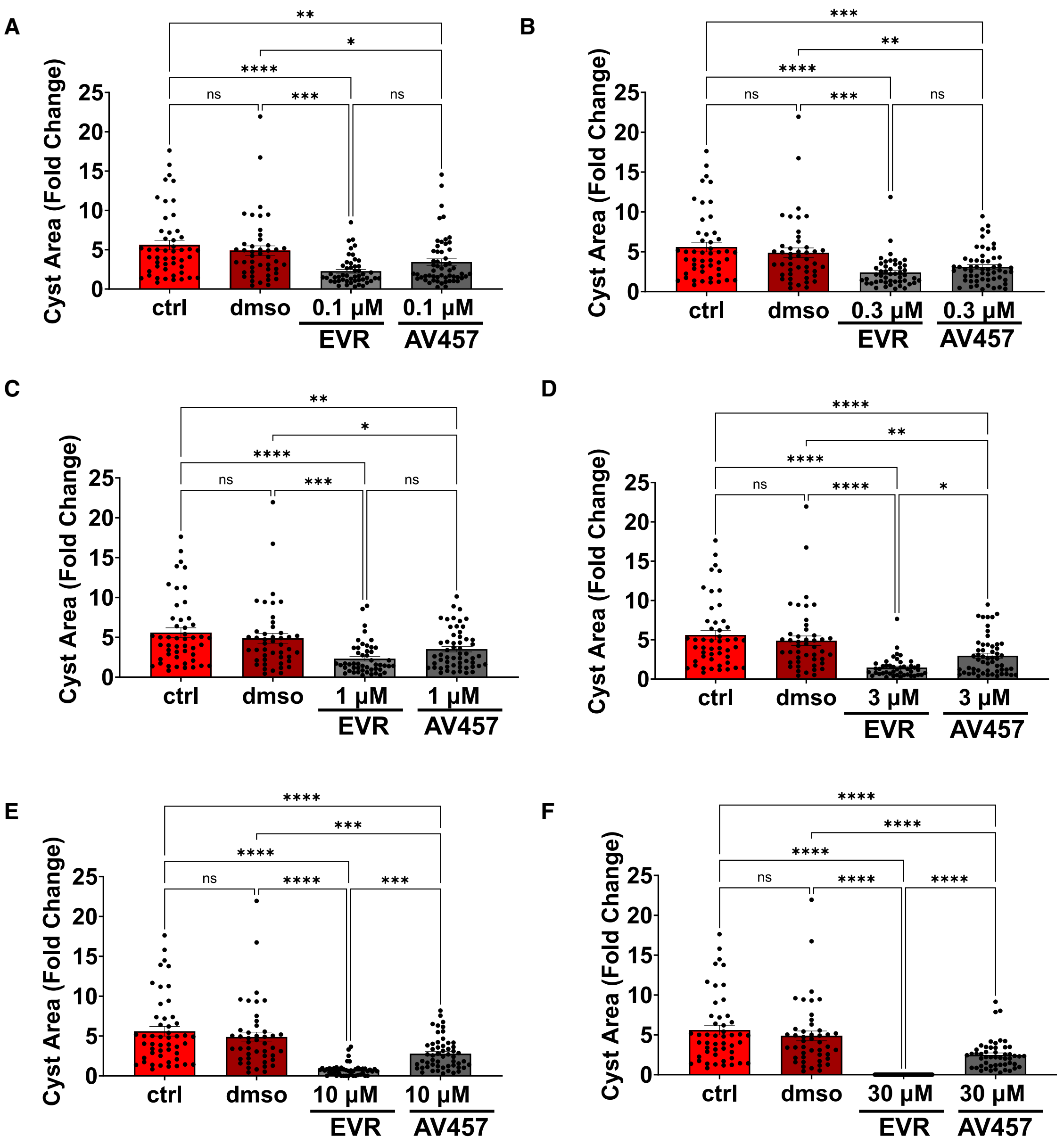

Figure 4. mTOR inhibitors exhibit similar efficacies at sub-toxic doses

Cyst area fold change measurements on day 14 for organoids treated with AV457 or everolimus (mean ± SEM; $n > 40$ organoids per condition pooled from 8 independent experiments, five with *PKD1*$^{-/-}$ and three with *PKD2*$^{-/-}$ organoids), with statistical analysis comparing everolimus (EVR) to AV457 (ns, not significant; *, $p \leq 0.05$; **, $p \leq 0.01$; ***, $p \leq 0.001$; ****, $p \leq 0.0001$ by one-way ANOVA), at treatment concentrations of (A) 0.1 µM, (B) 0.3 µM, (C) 1 µM, (D) 3 µM, (E) 10 µM, or (F) 30 µM.

supported the conclusion that everolimus exhibited significant cytotoxic effects within our dose range, which were not observed with AV457.

mTOR inhibitors show specific target engagement in PKD organoids

To verify that these compounds were achieving the proposed mechanism of action, we conducted immunoblot analysis of mTOR phosphorylation targets at different doses. Experiments in AV457 everolimus and control cultures were conducted side by side so that they could be readily compared. mTOR-specific phosphorylation of S6 (ribosomal protein S6, Ser240/244) and p70s6 (p70 ribosomal S6 kinase, Thr389) is a broad measure of mTOR inhibition that can be achieved with mTOR complex 1 inhibition alone. S6 and p70s6 phosphorylation events were

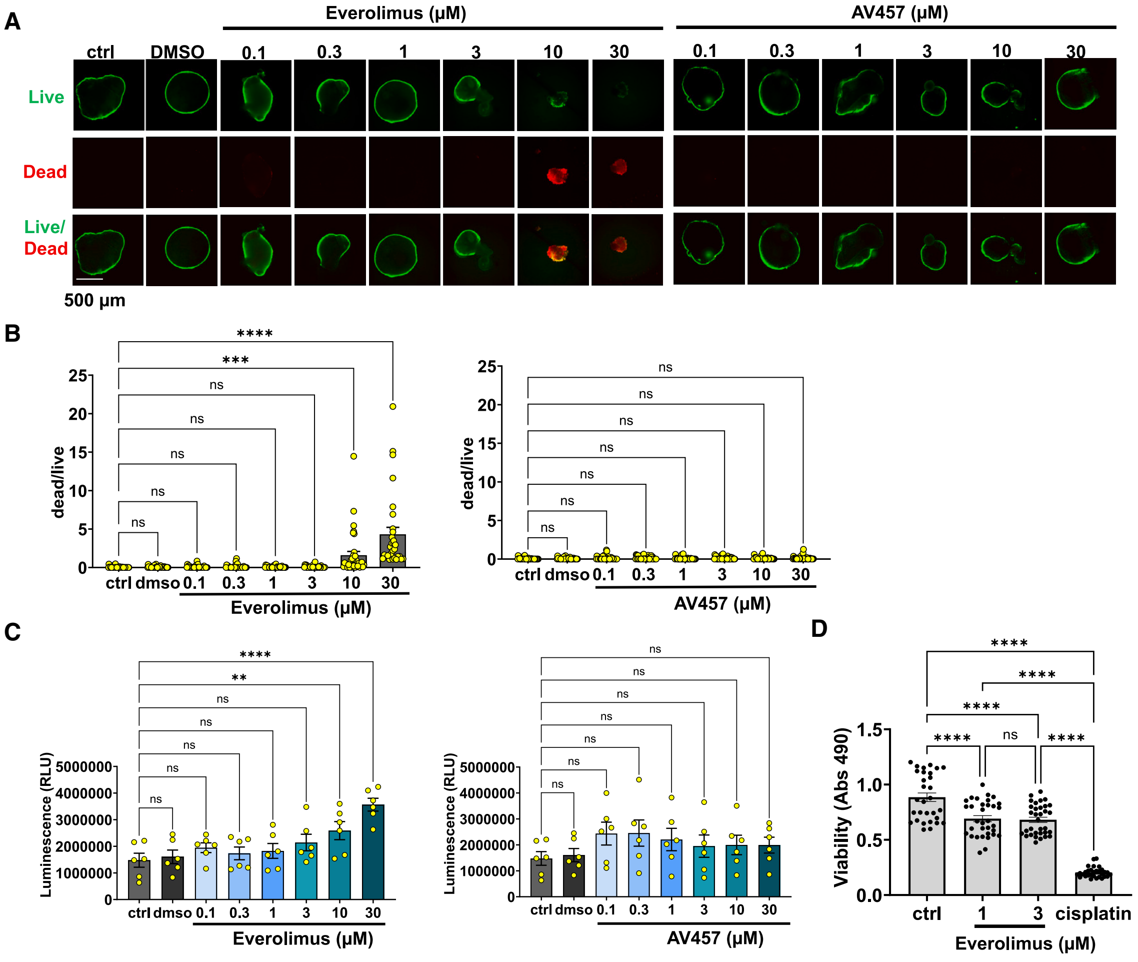

Figure 5. AV457 shows reduced toxicity relative to everolimus in PKD cysts

(A and B) (A) Representative confocal fluorescence images of live/dead staining on day 14 (scale bar, 500 μm) with (B) quantification using thresholding to measure area (mean ± SEM; $n > 30$ organoids per condition, pooled from 7 independent experiments—four using $PKD1^{-/-}$ and three using $PKD2^{-/-}$ organoids).

(C) LDH activity on day 7 (mean ± SEM; $n = 6$ independent experiments—three with $PKD1^{-/-}$ and three with $PKD2^{-/-}$ organoids; ns, not significant; *, $p \leq 0.05$; **, $p \leq 0.01$; ***, $p \leq 0.001$; ****, $p \leq 0.0001$).

(D) CellTiter-Glo assay in organoids following a 3-day treatment with everolimus or cisplatin (50 μM, positive control), compared to untreated control (ctrl) organoids (mean ± SEM; $n \geq 20$ wells per condition pooled from three independent experiments using human kidney organoids).

Statistical significance: ns, not significant; *, $p \leq 0.05$; **, $p \leq 0.01$; ***, $p \leq 0.001$; ****, $p \leq 0.0001$ by one-way ANOVA.

readily detected in control organoids but were rapidly abolished in mTOR inhibitor-treated organoids at doses ≥1 μM (Figures 6A and S6). This effect was statistically significant at all doses of both compounds, indicating efficient target engagement of mTORC1 (Figures 6B and 6C). Subsequently, we investigated phosphorylation of AKT (RAC-alpha serine/threonine protein kinase, Ser473), which is expected to be diminished in the setting of mTORC2 inhibition as part of a feedback mechanism (Figure 6D). While AKT phosphorylation was generally observed in all conditions, including untreated organoids, we observed a significant reduction in P-AKT at the 10 μM dose of everolimus, but not AV457 (Figures 6E, 6F, and S6). Thus, AV457 exhibited increased selectivity for mTORC1 in PKD organoids, which may contribute to its improved safety profile.

To assess AV457 selectivity over a wider dose range and in a different system, we analyzed S6 and AKT phosphorylation in PC3 (prostate cancer 3) cells, a human cell line derived from a prostate adenocarcinoma, which has been utilized previously to assess the selectivity of rapalogs

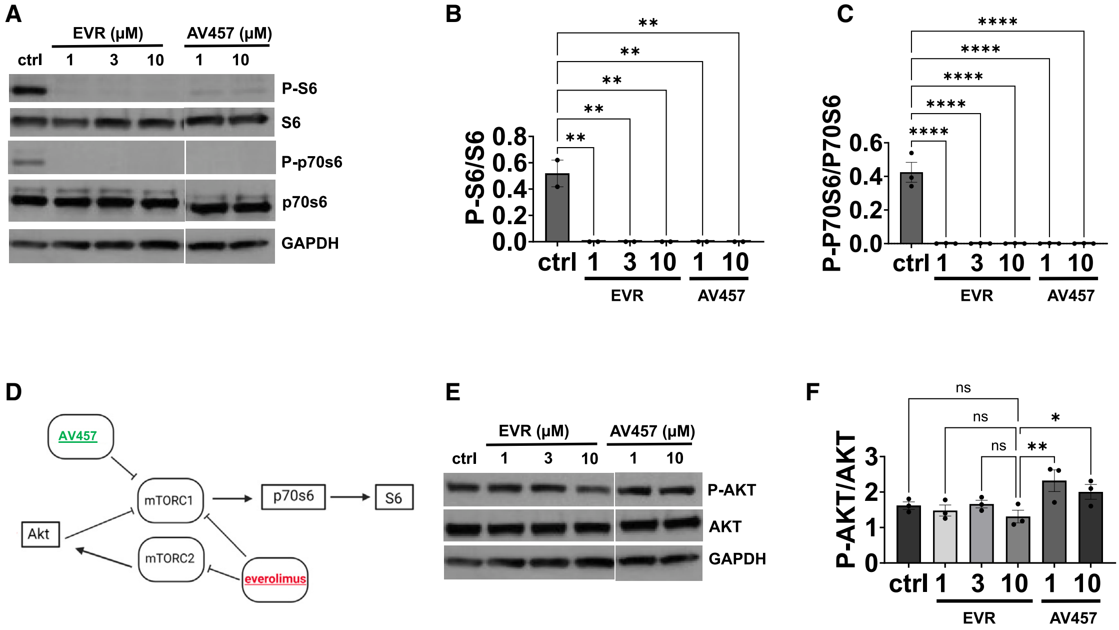

Figure 6. AV457 shows selective target engagement in PKD organoids

(A–C) (A) Representative western blots for mTOR signaling pathway components with (B) quantification of band intensities (mean ± SEM from 3 independent experiments for P-S6/S6, two with *PKD2*$^{-/-}$ and one with *PKD1*$^{-/-}$ organoids); (C) quantification for P-p70s6/p70s6 (mean ± SEM from 2 independent experiments, one with *PKD2*$^{-/-}$ and one with *PKD1*$^{-/-}$; ns, not significant; *, $p \leq 0.05$; **, $p \leq 0.01$; ***, $p \leq 0.001$; ****, $p \leq 0.0001$, by one-way ANOVA). GAPDH (glyceraldehyde-3-phosphate dehydrogenase) is shown as a loading control.

(D) Hypothetical diagram of the effect of compounds on the mTOR pathway.

(E) Representative western blots for AKT and P-AKT, with GAPDH loading control.

(F) Western blot analysis for P-AKT/AKT combined from *PKD2*$^{-/-}$ and *PKD1*$^{-/-}$ organoid batches (mean ± SEM; data pooled from 3 independent experiments, two with *PKD2*$^{-/-}$ and one with *PKD1*$^{-/-}$; ns, not significant; *, $p \leq 0.05$; **, $p \leq 0.01$; ***, $p \leq 0.001$; ****, $p \leq 0.0001$).

(Schreiber et al., 2019). While S6 phosphorylation was abolished by both everolimus and AV457, only everolimus resulted in a decrease in AKT phosphorylation, whereas AV457 treatment instead caused a gradual increase in P-AKT (Figures 7A–7D and S7). Such an increase can be observed as a consequence of mTORC1 inhibition (Breuleux et al., 2009; Vadlakonda et al., 2013). Thus the experiments suggested that AV457 was indeed more selective than everolimus and that this effect was generalizable between different types of cells.

DISCUSSION

Human organoids have several advantages over mouse models in these PKD assays. The organoid system is cost effective, requiring small quantities of the compound. The relative growth of cysts can be monitored simply by imaging with a transmitted light microscope. This enabled us to determine the safety and efficacy of two mTOR inhibitors in a dose-dependent and time-dependent manner. To model interventional treatment, we conducted these assays in organoids that had already begun to form cysts. Overall, we observed efficacy of both mTOR inhibitors tested within a safe dose range. The IC50 of both everolimus and AV457 was similar, in the range of previously established values for everolimus (Sedrani et al., 1998). In each case, efficacy increased cumulatively over time, which suggests that treating organoids prior to cyst formation would likely have even more dramatic effects.

In addition to efficacy, we observed toxicity of these compounds toward the higher concentration range. Everolimus was overtly toxic at 10 μM and 30 μM, whereas AV457 showed no evident toxicity even at 30 μM. For everolimus, a non-linear decline in PKD cyst growth was observed at the 3 μM dose, with reduced ATP levels, suggesting a subtle toxic effect. At the 1 μM dose, which showed no overt toxicity for either drug, the efficacy of everolimus and AV457 was similar. Target engagement was also comparable between AV457 and everolimus at the 1 μM concentration. We therefore favor the use of

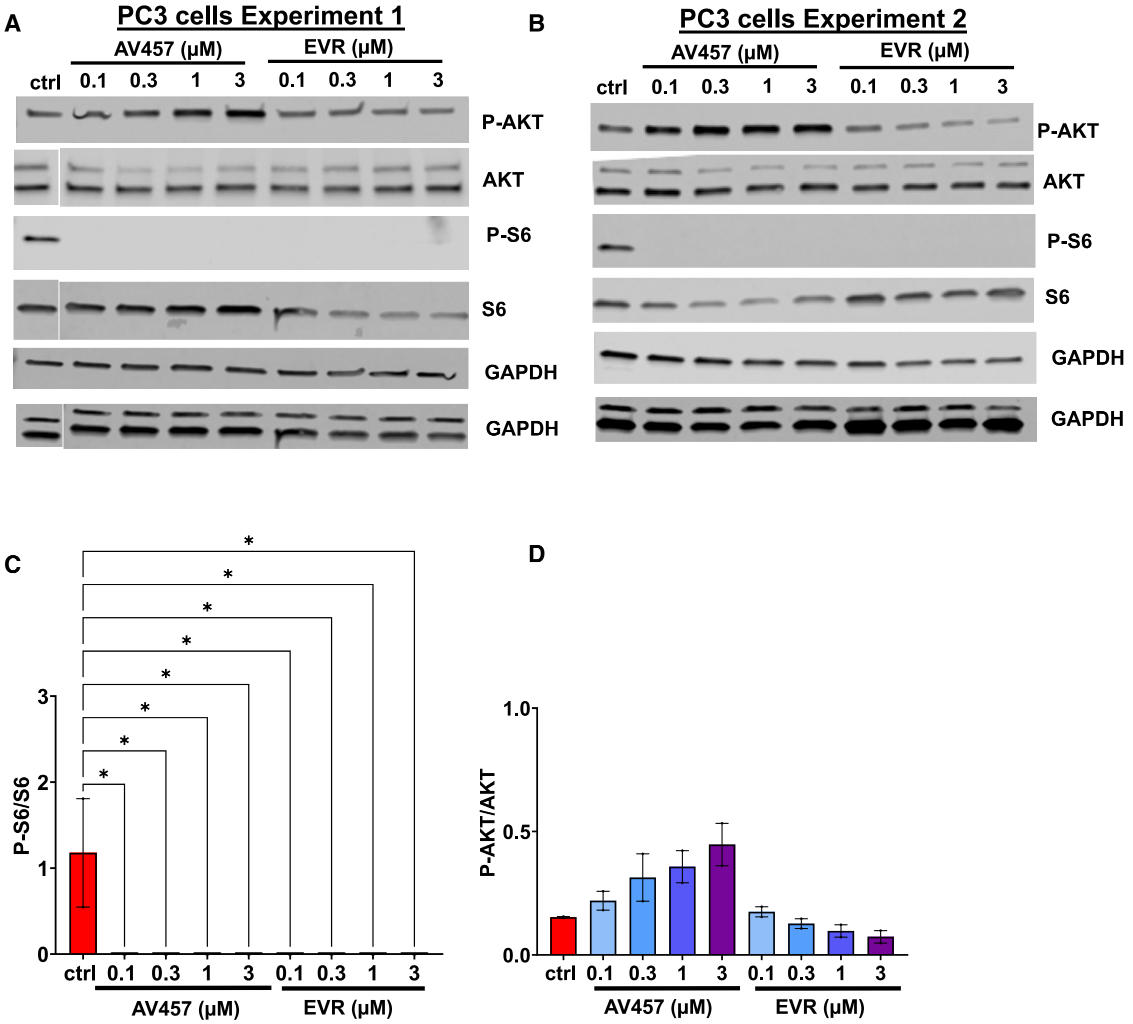

Figure 7. AV457 shows selective target engagement in PC3 cells

(A and B) Western blots for AKT, P-AKT, S6, and P-S6 in PC3 cells from two independent experiments, with (C) quantification of band intensities for P-S6/S6 and (D) P-AKT/AKT (mean ± SEM from 2 independent experiments; *, $p < 0.05$ by one-way ANOVA). For experiment 1, the blots for S6, AKT, and their corresponding GAPDH initially had the control sample at the end of the blot. For consistency, these were cropped and repositioned with the control sample at the beginning, while the other blots remained unchanged.

AV457 as opposed to everolimus within this safer dose range, as AV457 demonstrated a superior safety profile in our assays.

In a previous clinical trial for PKD, everolimus treatment over a two-year period reduced total kidney volume but failed to achieve a statistical difference in glomerular filtration rate (Walz et al., 2010). Everolimus was administered to achieve a trough level of ~5 nM, which may have limited its efficacy (Walz et al., 2010). Nevertheless, the everolimus arm of the trial was associated with side effects typical of mTOR inhibition, which would preclude administration of a higher dose. In our PKD organoids, we tested doses of mTOR inhibitors that were considerably higher than those in the preceding clinical trial, to account for the more rapid time course of disease and therapy in this model. Our efficacious doses, however, were lower than the dose of 10 μM for rapamycin suggested in a preliminary analysis of chemicals for PKD, which did not account for potential toxicity (Tran et al., 2022).

To demonstrate reproducibility, we have conducted experiments in both *PKD1* and *PKD2* mutants, consistent with previous studies (Freedman et al., 2015; Cruz et al., 2017; Freedman 2022; Pamies et al., 2024; Vishy et al., 2024). Responses of both efficacy and cytotoxicity were observed in both genotypes. As expected when utilizing distinct cell lines, some differences were also observed, which may reflect subtle differences between the responses of *PKD1* versus *PKD2* mutants, or alternatively non-specific

differences between cell lines and batches (Freedman et al., 2015; Cruz et al., 2017; Freedman 2022; Pamies et al., 2024; Vishy et al., 2024). In addition to human kidney organoids, we also utilized PC3 cells to demonstrate the selectivity of AV457, which is a new candidate therapeutic.

Examining AV457 for the first time, our data indicate that AV457 provides a more favorable balance of safety and efficacy for PKD compared to everolimus and merit consideration as investigational therapeutics in human clinical trials, compared to other drugs (Yu et al., 2015). Organoids appear well positioned to inform early dosing and proof-of-concept evaluations for compounds such as AV457 and other rapalogs, which can be compared to standard therapeutics used previously in clinical trials before they can progress into the next stage of drug development.

EXPERIMENTAL PROCEDURES

Kidney organoid differentiation

PKD2$^{-/-}$ (exon 1 null mutant, gRNA CGTGGAGCCGC GATAACCC, or R186X null mutant, gRNA CCCGA GTGGCCTGGGCGGAG) or *PKD1*$^{-/-}$ (Q3838X exon 41 mutant, gRNA CGTGGAGCCGCGATAACCC) iPS cells derived using CRISPR base editing from the parental WTC-11 cell line (Coriell GM25256) were dissociated with Accutase (STEMCELL Technologies) and plated in mTeSR1 (STEMCELL Technologies) supplemented with Y-27632 dihydrochloride (Tocris) at 3,000, 4,000, and 5,000 cells/well in 24-well plates (MilliporeSigma) pre-coated overnight with 300 μL/well of DMEM-F12 (Thermo Fisher Scientific) containing 1% Geltrex (Thermo Fisher Scientific), then changed to mTeSR1 + 1.5% Geltrex after 24 h and mTeSR1 24 h later (500 μL/well). 24 h later, media were changed to Advanced RPMI (Thermo Fisher Scientific) + 2% Pen/Strep (Thermo Fisher Scientific) + 1% GlutaMAX (Thermo Fisher Scientific) + 12 μM CHIR99021 (ReproCELL) for 36 h, and then to RB (ARPMI-B27, comprising Advanced RPMI + 2% Pen/Strep + 1% GlutaMAX + B27 [Thermo Fisher Scientific]) replaced every other day until day 21. Studies utilizing human pluripotent stem cells were conducted with the approval of the University of Washington's Embryonic Stem Cell Research Oversight Committee.

Treatments

On the day after seeding, organoids were picked using a 23-gauge needle from all wells and transferred into a low-attachment 6-well plate (Corning) in RB media and changed every 2 days, until day 30. Using a P200 pipetteman, individual cystic organoids were transferred into a low-attachment 96-well plate (Corning), 9 organoids per row. Conditions were untreated or treated with 0.5% DMSO (Thermo Fisher Scientific, vehicle control), AV457, or everolimus (provided by Aeovian Pharmaceuticals). Compounds were first serially diluted in series in DMSO to achieve 6 different stock concentrations and subsequently these were diluted 1:200 into media to achieve 6 corresponding final concentrations (0.1, 0.3, 1, 3, 10, and 30 μM). Media with fresh compound were changed every other day except the weekends.

PC3 cells

Cells (ATCC-CRL-1435) were plated at 100,000 cells per well in a 24-well plate and grown in Kaigh's Modification of Ham's F-12 Medium supplemented with 10 % FBS until they approached confluency. The cells were then treated with everolimus and AV457 at concentrations of 0.1, 0.3, 1, and 3 μM for 48 h (three wells per treatment). Following treatment, the cells were lysed and western blot analysis was performed.

Phase-contrast imaging and analysis

Each organoid was imaged immediately prior to treatment (day 0) and on days 1, 3, 7, and 14. All images were opened simultaneously in ImageJ, region of interest was drawn manually around the cysts, area measurements were recorded, and cyst fold change was calculated (Microsoft Excel, dividing each day consecutively day by day 0). Subsequently, linear and bar graphs of the normalized fold change data were generated (GraphPad Prism 10.3.1).

LDH assay

LDH activity was measured on days 3, 7, and 14 using LDH-Glo cytotoxicity assay (Promega). 25 μL of the media was transferred to a clear-bottom white 96-well plate (Greiner Bio-One), 25 μL mixture of LDH detection enzyme mix with reductase substrate was added, and the plate was incubated for 1 h at room temperature. Luminescence assay was run on Precisely plate reader (PerkinElmer). An average of each treatment condition ($n \geq 8$ organoids per condition) was calculated. These averages were pooled and graphed using GraphPad Prism 10.

Viability assay

Live/dead assay was done on day 14 of treatment. To a total of 3,500 μL of RB media, we added 5 μL of Calcein AM (Life Technologies) stock (1 mg/mL) and 5 μL of propidium iodide (Thermo Fisher Scientific) stock (1 mg/mL). This mixture was added 1:1 to the well, incubated for 30 min, and a single z slice through the center of the organoid was imaged at 10x on a confocal live cell imaging scope.

CellTiter-Glo assay

The CellTiter 96 AQ_{ueous} One Solution assay (Promega-G358A) was performed after organoids were pretreated

for 3 days with everolimus (1 μM and 3 μM) or cisplatin compared to an untreated control. The media were changed on day 2. Two experiments were conducted starting on day 21 with organoids grown in a 96-well plate (*PKD1*$^{-/-}$). A third experiment commenced on day 22, using organoids grown in a 96-well plate (WTC-11 background). The assay was executed by mixing 20 μL of CellTiter 96 reagent with 100 μL of DMEM/F12 medium. After removing the culture medium from the wells, the CellTiter 96-DMEM/F12 mixture (100 μL) was added to each well. The plates were incubated in a tissue culture incubator at 37°C for 1 h. Absorbance readings were then taken at a wavelength of 492 nm using a Precisely plate reader (PerkinElmer).

Western blot

After treatment, 24-well plate was washed with PBS and lysed with radioimmunoprecipitation assay lysis buffer containing 7x protease inhibitor (Sigma-Aldrich) and 20x phosphotase inhibitor (Roche) with 0.3 μL of Benzonase nuclease (Sigma-Aldrich). After, 5 min incubation, lysate was centrifuged at 16,000 × *g* for 15 min at 4°C. The protein content was measured using bicinchoninic acid protein assay kit (Thermo Fisher Scientific). The samples were boiled for 5 min at 95°C in 1x Laemmli sample buffer (Bio-Rad). Proteins were then separated using 4%–20% gel (Bio-Rad) and transferred onto a nitrocellulose membrane. The membrane was blocked for 1 h using 5% milk with 1x Tris-buffered saline (TBS) plus 1% Tween 20 (TBST) and then incubated in primary antibody at 4°C overnight with gentle shaking. The next day, the membrane was washed using TBST 3 times with intervals of 5, 10, and 15 min. The secondary was added for 1 h at room temperature. Then, another three washes were performed. The membrane was then incubated for 1 min in 2 mL of Pierce ECL western blotting substrate (Thermo Fisher Scientific) and developed using enhanced chemiluminescence blotting film (Genesee Scientific). The analysis was performed using Fiji ImageJ. The antibodies used were AKT (40D4) (Cell Signaling Technology), P-AKT (S473) (Cell Signaling Technology), p70 S6 (49D7) (Cell Signaling Technology), P-p70 S6 (T389) (Cell Signaling Technology), S6 (5G10) (Cell Signaling Technology), and P-S6 (S240/244) (Cell Signaling Technology).

Statistical analysis

All measurements were transferred into GraphPad Prism 10. Ordinary one-way ANOVA was used to measure the significant difference between columns for data that were presented as bar graphs. Two-way ANOVA was performed to evaluate the effects of dose and treatment time on cyst fold change for each compound. When data were missing, a mixed-effects model was applied to handle incomplete datasets. Dose-response data for each compound (everolimus and AV457) were used to calculate IC50 values, utilizing non-linear regression with a four-parameter logistic model.

Structures

Structures of chemicals were generated using the Chemical Sketch Tool (https://www.rcsb.org/chemical-sketch).

RESOURCE AVAILABILITY

Lead contact

Requests for further information, materials, raw data, and resources should be directed to and will be fulfilled by the lead contact, Benjamin S. Freedman (benof@uw.edu).

Materials availability

Samples of reagents generated or utilized in this study are available from the lead contact with completed materials transfer agreements. Limited quantities of AV457 are available. Cell lines may require additional agreements with third parties holding rights to the materials. Reasonable compensation may be charged for processing and shipping to produce, package, and maintain the materials.

Data and code availability

All data points are shown in the figures. Raw data are maintained at the institution and may be provided for re-analysis upon reasonable request. Data may be subject to additional agreements with third parties. No specialized code was developed for this project.

ACKNOWLEDGMENTS

We thank John Kincaid, Annica Martensson, and Allison Hulme (Aeovian Pharmaceuticals) for helpful discussions and for supplying everolimus and AV457 for these experiments. We thank Jonathan Himmelfarb (Icahn School of Medicine at Mount Sinai) and Hongxia Fu (University of Washington and Plurexa) for helpful discussions. The work was supported by a research grant from Aeovian Pharmaceuticals (B.S.F.); NIH awards R01DK117914 (B.S.F.), U2CTR004867 (B.S.F.), R41DK136452 (Fu and B.S.F.), and UG3TR003288 (Himmelfarb); a Washington Research Foundation Technology Commercialization grant (B.S.F.); and the Lara Nowak Macklin Research Fund.

AUTHOR CONTRIBUTIONS

B.S.F. conceived the study. B.S.F., R.E.G., and P.A. designed the experiments. R.E.G. and P.A. performed the experiments. B.S.F., R.E.G., and P.A. analyzed the data. B.S.F., R.E.G., and P.A. wrote the manuscript.

DECLARATION OF INTERESTS

B.S.F. is an inventor on patents and/or patent applications related to human kidney organoid differentiation and modeling of PKD in this system (these include "Three-dimensional differentiation of epiblast spheroids into kidney tubular organoids modeling human microphysiology, toxicology, and morphogenesis" [Japan, US, and

Australia], licensed to STEMCELL Technologies; “High-throughput automation of organoids for identifying therapeutic strategies” [PTC patent application pending]; and “Systems and methods for characterizing pathophysiology” [PTC patent application pending]). B.S.F. has ownership interest in Plurexa LLC. None of the preceding interests affected in any way the results of the paper or would be affected by them, but they are shared by way of transparency.

SUPPLEMENTAL INFORMATION

Supplemental information can be found online at https://doi.org/10.1016/j.stemcr.2024.102395.

Received: March 11, 2024
Revised: December 20, 2024
Accepted: December 21, 2024
Published: January 23, 2025

REFERENCES

Arriola Apelo, S.I., Neuman, J.C., Baar, E.L., Syed, F.A., Cummings, N.E., Brar, H.K., Pumper, C.P., Kimple, M.E., and Lamming, D.W. (2016). Alternative rapamycin treatment regimens mitigate the impact of rapamycin on glucose homeostasis and the immune system. Aging Cell *15*, 28–38.

Breuleux, M., Klopfenstein, M., Stephan, C., Doughty, C.A., Barys, L., Maira, S.M., Kwiatkowski, D., and Lane, H.A. (2009). Increased AKT S473 phosphorylation after mTORC1 inhibition is rictor dependent and does not predict tumor cell response to PI3K/mTOR inhibition. Mol. Cancer Therapeut. *8*, 742–753.

Canaud, G., Knebelmann, B., Harris, P.C., Vrtovsnik, F., Correas, J.M., Pallet, N., Heyer, C.M., Letavernier, E., Bienaimé, F., Thervet, E., et al. (2010). Therapeutic mTOR inhibition in autosomal dominant polycystic kidney disease: What is the appropriate serum level? Am. J. Transplant. *10*, 1701–1706.

Cruz, N.M., Song, X., Czerniecki, S.M., Gulieva, R.E., Churchill, A.J., Kim, Y.K., Winston, K., Tran, L.M., Diaz, M.A., Fu, H., et al. (2017). Organoid cystogenesis reveals a critical role of microenvironment in human polycystic kidney disease. Nat. Mater. *16*, 1112–1119.

Czerniecki, S.M., Cruz, N.M., Harder, J.L., Menon, R., Annis, J., Otto, E.A., Gulieva, R.E., Islas, L.V., Kim, Y.K., Tran, L.M., et al. (2018). High-Throughput Screening Enhances Kidney Organoid Differentiation from Human Pluripotent Stem Cells and Enables Automated Multidimensional Phenotyping. Cell Stem Cell *22*, 929–940.e4.

Freedman, B.S. (2022). Physiology assays in human kidney organoids. Am. J. Physiol. Ren. Physiol. *322*, F625–F638.

Freedman, B.S., Brooks, C.R., Lam, A.Q., Fu, H., Morizane, R., Agrawal, V., Saad, A.F., Li, M.K., Hughes, M.R., Werff, R.V., et al. (2015). Modelling kidney disease with CRISPR-mutant kidney organoids derived from human pluripotent epiblast spheroids. Nat. Commun. *6*, 8715.

Jardine, M.J., Liyanage, T., Buxton, E., and Perkovic, V. (2013). mTOR inhibition in autosomal-dominant polycystic kidney disease (ADPKD): the question remains open. Nephrol. Dial. Transplant. *28*, 242–244.

Lamming, D.W., Ye, L., Katajisto, P., Goncalves, M.D., Saitoh, M., Stevens, D.M., Davis, J.G., Salmon, A.B., Richardson, A., Ahima, R.S., et al. (2012). Rapamycin-induced insulin resistance is mediated by mTORC2 loss and uncoupled from longevity. Science *335*, 1638–1643.

Li, S.R., Gulieva, R.E., Helms, L., Cruz, N.M., Vincent, T., Fu, H., Himmelfarb, J., and Freedman, B.S. (2022). Glucose absorption drives cystogenesis in a human organoid-on-chip model of polycystic kidney disease. Nat. Commun. *13*, 7918.

Neufeld, T.K., Douglass, D., Grant, M., Ye, M., Silva, F., Nadasdy, T., and Grantham, J.J. (1992). In vitro formation and expansion of cysts derived from human renal cortex epithelial cells. Kidney Int. *41*, 1222–1236.

Pamies, D., Ekert, J., Zurich, M.G., Frey, O., Werner, S., Piergiovanni, M., Freedman, B.S., Keong Teo, A.K., Erfurth, H., Reyes, D.R., et al. (2024). Recommendations on fit-for-purpose criteria to establish quality management for microphysiological systems and for monitoring their reproducibility. Stem Cell Rep. *19*, 1041–1617.

Sarbassov, D.D., Ali, S.M., Kim, D.H., Guertin, D.A., Latek, R.R., Erdjument-Bromage, H., Tempst, P., and Sabatini, D.M. (2004). Rictor, a novel binding partner of mTOR, defines a rapamycin-insensitive and raptor-independent pathway that regulates the cytoskeleton. Curr. Biol. *14*, 1296–1302.

Schreiber, K.H., Arriola Apelo, S.I., Yu, D., Brinkman, J.A., Velarde, M.C., Syed, F.A., Liao, C.Y., Baar, E.L., Carbajal, K.A., Sherman, D.S., et al. (2019). A novel rapamycin analog is highly selective for mTORC1 *in vivo*. Nat. Commun. *10*, 3194.

Sedrani, R., Cottens, S., Kallen, J., and Schuler, W. (1998). Chemical modification of rapamycin: the discovery of SDZ RAD. Transplant. Proc. *30*, 2192–2194.

Serra, A.L., Poster, D., Kistler, A.D., Krauer, F., Raina, S., Young, J., Rentsch, K.M., Spanaus, K.S., Senn, O., Kristanto, P., et al. (2010). Sirolimus and kidney growth in autosomal dominant polycystic kidney disease. N. Engl. J. Med. *363*, 820–829.

Shillingford, J.M., Murcia, N.S., Larson, C.H., Low, S.H., Hedgepeth, R., Brown, N., Flask, C.A., Novick, A.C., Goldfarb, D.A., Kramer-Zucker, A., et al. (2006). The mTOR pathway is regulated by polycystin-1, and its inhibition reverses renal cystogenesis in polycystic kidney disease. Proc. Natl. Acad. Sci. USA *103*, 5466–5471.

Torres, V.E., Gansevoort, R.T., and Czerwiec, F.S. (2018). Tolvaptan in Later-Stage Polycystic Kidney Disease. N. Engl. J. Med. *378*, 489–490.

Tran, T., Song, C.J., Nguyen, T., Cheng, S.Y., McMahon, J.A., Yang, R., Guo, Q., Der, B., Lindström, N.O., Lin, D.C.H., and McMahon, A.P. (2022). A scalable organoid model of human autosomal dominant polycystic kidney disease for disease mechanism and drug discovery. Cell Stem Cell *29*, 1083–1101.e7.

Trelinska, J., Dachowska, I., Kotulska, K., Fendler, W., Jozwiak, S., and Mlynarski, W. (2015). Complications of mammalian target of rapamycin inhibitor anticancer treatment among patients

with tuberous sclerosis complex are common and occasionally life-threatening. Anti Cancer Drugs *26*, 437–442.

Vadlakonda, L., Dash, A., Pasupuleti, M., Anil Kumar, K., and Reddanna, P. (2013). The Paradox of Akt-mTOR Interactions. Front. Oncol. *3*, 165.

Vishy, C.E., Thomas, C., Vincent, T., Crawford, D.K., Goddeeris, M.M., and Freedman, B.S. (2024). Genetics of cystogenesis in base-edited human organoids reveal therapeutic strategies for polycystic kidney disease. Cell Stem Cell *31*, 537–553.e5.

Wahl, P.R., Serra, A.L., Le Hir, M., Molle, K.D., Hall, M.N., and Wüthrich, R.P. (2006). Inhibition of mTOR with sirolimus slows disease progression in Han:SPRD rats with autosomal dominant polycystic kidney disease (ADPKD). Nephrol. Dial. Transplant. *21*, 598–604.

Walz, G., Budde, K., Mannaa, M., Nürnberger, J., Wanner, C., Sommerer, C., Kunzendorf, U., Banas, B., Hörl, W.H., Obermüller, N., et al. (2010). Everolimus in patients with autosomal dominant polycystic kidney disease. N. Engl. J. Med. *363*, 830–840.

Wu, M., Arcaro, A., Varga, Z., Vogetseder, A., Le Hir, M., Wüthrich, R.P., and Serra, A.L. (2009). Pulse mTOR inhibitor treatment effectively controls cyst growth but leads to severe parenchymal and glomerular hypertrophy in rat polycystic kidney disease. Am. J. Physiol. Ren. Physiol. *297*, F1597–F1605.

Xu, Y., Kuppe, C., Perales-Patón, J., Hayat, S., Kranz, J., Abdallah, A.T., Nagai, J., Li, Z., Peisker, F., Saritas, T., et al. (2022). Adult human kidney organoids originate from CD24(+) cells and represent an advanced model for adult polycystic kidney disease. Nat. Genet. *54*, 1690–1701.

Yao, J.C., Fazio, N., Singh, S., Buzzoni, R., Carnaghi, C., Wolin, E., Tomasek, J., Raderer, M., Lahner, H., Voi, M., et al. (2016). Everolimus for the treatment of advanced, non-functional neuroendocrine tumours of the lung or gastrointestinal tract (RADIANT-4): a randomised, placebo-controlled, phase 3 study. Lancet *387*, 968–977.

Yu, Alan S.L., El-Ters, M., and Winklhofer, F.T. (2015). Clinical trials in autosomal dominant polycystic kidney disease (Exon Publications), pp. 109–135.

Stem Cell Reports, Volume *20*

Supplemental Information

A novel rapalog shows improved safety vs. efficacy in a human organoid model of polycystic kidney disease

Ramila E. Gulieva, Parvaneh Ahmadvand, and Benjamin S. Freedman

SUPPLEMENTAL FIGURE S1

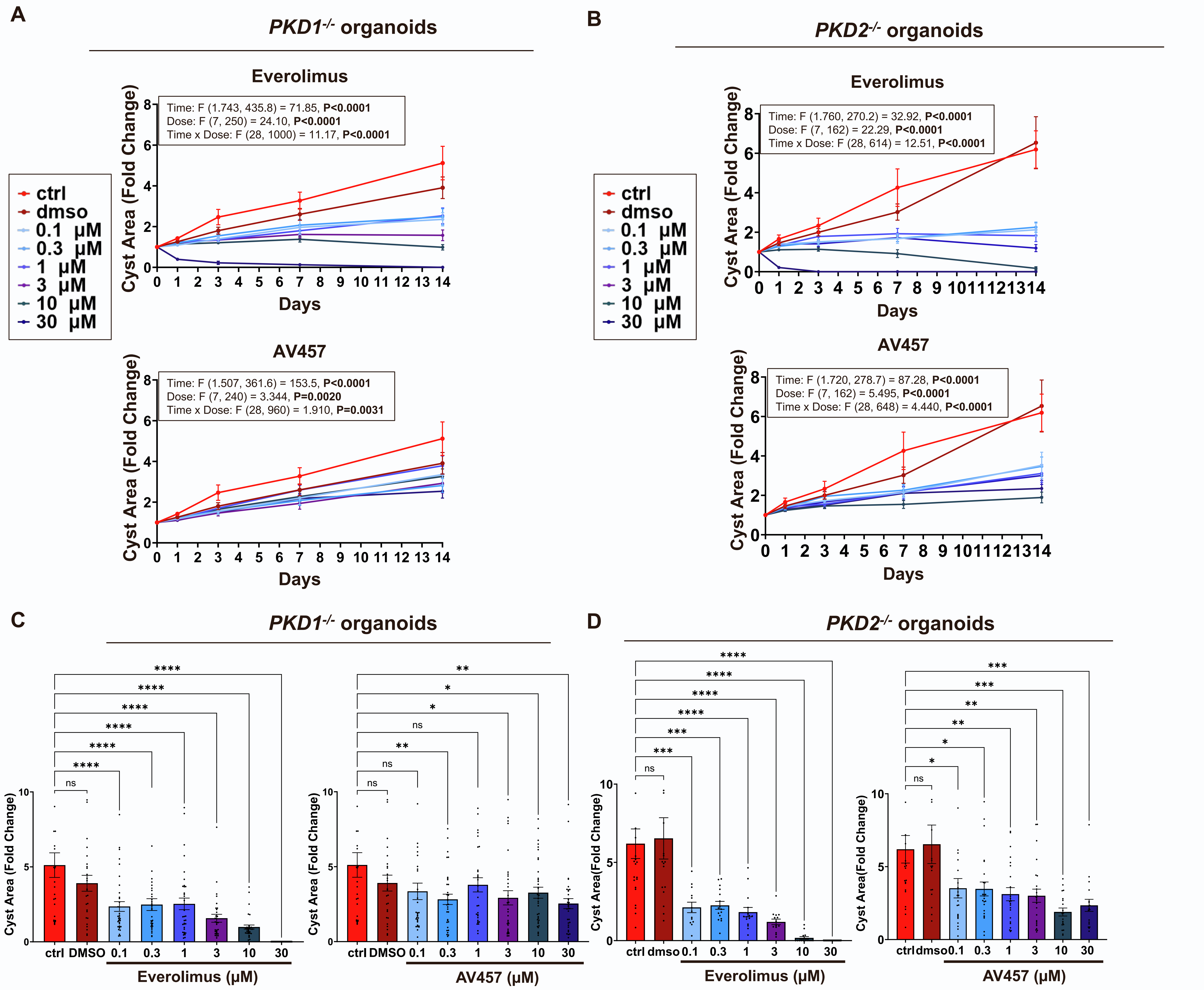

Supplemental Figure S1. mTOR inhibitors reduce cyst growth in *PKD1*$^{-/-}$ and *PKD2*$^{-/-}$ organoids. (A) Cyst area fold change measurements for organoids treated with AV457 or everolimus (mean ± s.e.m.; n > 40 organoids per condition, pooled from 5 independent experiments using *PKD1*$^{-/-}$ organoids. Boxes show two-way ANOVA analysis for Time and Dose dependence). (B) Cyst area fold change measurements from 3 independent experiments using *PKD2*$^{-/-}$ organoids. (C) Detailed analysis of cyst area fold change on day 14 for *PKD1*$^{-/-}$, with statistical analysis, and (D) corresponding analysis for *PKD2*$^{-/-}$ organoids. Statistical significance is indicated as follows: ns, not significant; *p ≤ 0.05; **p ≤ 0.01; ***p ≤ 0.001; ****p ≤ 0.0001.

SUPPLEMENTAL FIGURE S2

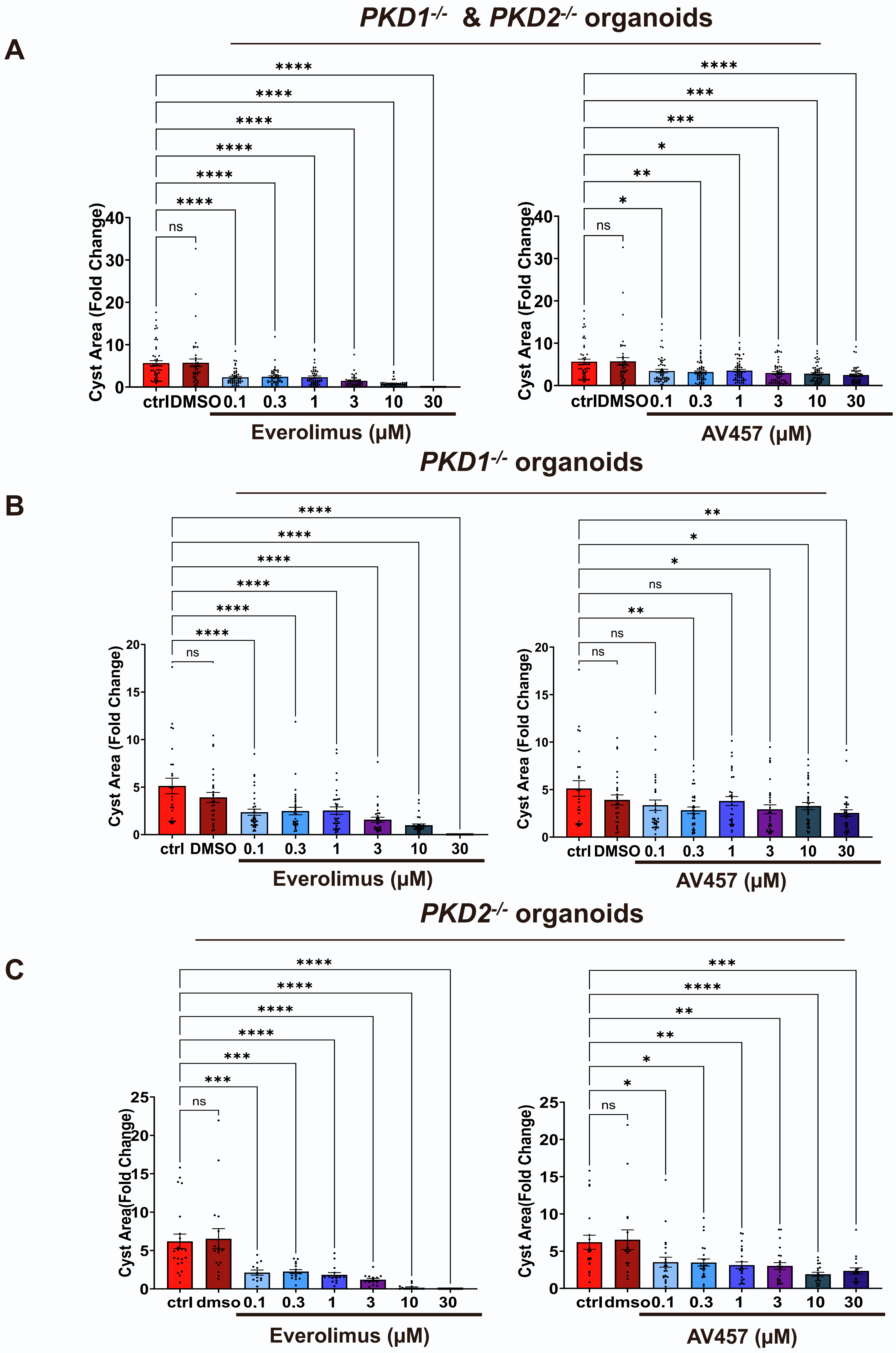

Supplementary Figure S2. mTOR inhibitors reduce PKD cyst growth over time. (A) Full-range data for both *PKD1*$^{-/-}$ and *PKD2*$^{-/-}$ organoids from Figure 2D. (B) Full-range data for *PKD1*$^{-/-}$ organoids from Supplementary Figure S1C and (C) for *PKD2*$^{-/-}$ organoids from Supplementary Figure S1D. Statistical significance is indicated as follows: ns, not significant; *p ≤ 0.05; **p ≤ 0.01; ***p ≤ 0.001; ****p ≤ 0.0001.

SUPPLEMENTAL FIGURE S3

A

PKD1$^{-/-}$ & *PKD2*$^{-/-}$ organoids

Everolimus

Tukey's multiple comparisons test	Mean Diff.	Summary	Adjusted P Value
0.1 vs. 0.3	-0.1139	ns	>0.9999
0.1 vs. 1	-0.02167	ns	>0.9999
0.1 vs. 3	0.8368	ns	0.8372
0.1 vs. 10	1.556	ns	0.1145
**0.1 vs. 30**	**2.293**	**	**0.0011**
0.3 vs. 1	0.0922	ns	>0.9999
0.3 vs. 3	0.9507	ns	0.727
0.3 vs. 10	1.669	ns	0.0679
**0.3 vs. 30**	**2.407**	***	**0.0005**
1 vs. 3	0.8585	ns	0.8145
1 vs. 10	1.577	ns	0.1004
**1 vs. 30**	**2.315**	***	**0.0009**
3 vs. 10	0.7188	ns	0.918
3 vs. 30	1.456	ns	0.1668
10 vs. 30	0.7375	ns	0.8845

AV457

Tukey's multiple comparisons test	Mean Diff.	Summary	Adjusted P Value
0.1 vs. 0.3	0.2561	ns	0.9999
0.1 vs. 1	-0.08259	ns	>0.9999
0.1 vs. 3	0.4638	ns	0.9948
0.1 vs. 10	0.6424	ns	0.9659
0.1 vs. 30	0.9539	ns	0.7855
0.3 vs. 1	-0.3387	ns	0.9993
0.3 vs. 3	0.2078	ns	>0.9999
0.3 vs. 10	0.3863	ns	0.9984
0.3 vs. 30	0.6978	ns	0.9497
1 vs. 3	0.5464	ns	0.9861
1 vs. 10	0.725	ns	0.9353
1 vs. 30	1.036	ns	0.7049
3 vs. 10	0.1785	ns	>0.9999
3 vs. 30	0.49	ns	0.9935
10 vs. 30	0.3115	ns	0.9997

B

PKD1$^{-/-}$ organoids

Everolimus

Tukey's multiple comparisons test	Mean Diff.	Summary	Adjusted P Value
0.1 vs. 0.3	-0.1271	ns	>0.9999
0.1 vs. 1	-0.176	ns	>0.9999
0.1 vs. 3	0.7831	ns	0.824
0.1 vs. 10	1.37	ns	0.1504
**0.1 vs. 30**	**2.356**	***	**0.0001**
0.3 vs. 1	-0.04892	ns	>0.9999
0.3 vs. 3	0.9101	ns	0.7072
0.3 vs. 10	1.497	ns	0.0973
**0.3 vs. 30**	**2.483**	****	**<0.0001**
1 vs. 3	0.9591	ns	0.631
1 vs. 10	1.546	ns	0.068
**1 vs. 30**	**2.532**	****	**<0.0001**
3 vs. 10	0.5869	ns	0.958
3 vs. 30	1.573	ns	0.0552
10 vs. 30	0.9863	ns	0.5229

AV457

Tukey's multiple comparisons test	Mean Diff.	Summary	Adjusted P Value
0.1 vs. 0.3	0.5346	ns	0.9936
0.1 vs. 1	-0.4351	ns	0.9982
0.1 vs. 3	0.4311	ns	0.9984
0.1 vs. 10	0.08955	ns	>0.9999
0.1 vs. 30	0.8178	ns	0.9271
0.3 vs. 1	-0.9697	ns	0.843
0.3 vs. 3	-0.1035	ns	>0.9999
0.3 vs. 10	-0.445	ns	0.9977
0.3 vs. 30	0.2833	ns	0.9999
1 vs. 3	0.8662	ns	0.9103
1 vs. 10	0.5247	ns	0.9935
1 vs. 30	1.253	ns	0.5794
3 vs. 10	-0.3416	ns	0.9996
3 vs. 30	0.3867	ns	0.9992
10 vs. 30	0.7283	ns	0.9551

C

PKD2$^{-/-}$ organoids

Everolimus

Tukey's multiple comparisons test	Mean Diff.	Summary	Adjusted P Value
0.1 vs. 0.3	-0.1313	ns	>0.9999
0.1 vs. 1	0.3012	ns	>0.9999
0.1 vs. 3	0.9255	ns	0.9867
0.1 vs. 10	1.949	ns	0.5538
0.1 vs. 30	2.123	ns	0.3059
0.3 vs. 1	0.4325	ns	0.9998
0.3 vs. 3	1.057	ns	0.9626
0.3 vs. 10	2.08	ns	0.3968
0.3 vs. 30	2.254	ns	0.1656
1 vs. 3	0.6243	ns	0.9985
1 vs. 10	1.648	ns	0.7086
1 vs. 30	1.822	ns	0.4467
3 vs. 10	1.024	ns	0.9711
3 vs. 30	1.197	ns	0.888
10 vs. 30	0.1735	ns	>0.9999

AV457

Tukey's multiple comparisons test	Mean Diff.	Summary	Adjusted P Value
0.1 vs. 0.3	0.04958	ns	>0.9999
0.1 vs. 1	0.4079	ns	0.9998
0.1 vs. 3	0.513	ns	0.9991
0.1 vs. 10	1.63	ns	0.6799
0.1 vs. 30	1.173	ns	0.927
0.3 vs. 1	0.3583	ns	>0.9999
0.3 vs. 3	0.4634	ns	0.9995
0.3 vs. 10	1.58	ns	0.7032
0.3 vs. 30	1.123	ns	0.9385
1 vs. 3	0.1051	ns	>0.9999
1 vs. 10	1.222	ns	0.9039
1 vs. 30	0.7649	ns	0.9934
3 vs. 10	1.117	ns	0.9355
3 vs. 30	0.6598	ns	0.9972
10 vs. 30	-0.4568	ns	0.9998

Supplementary Figure S3. Statistical summary for cyst fold change in *PKD1*$^{-/-}$ and *PKD2*$^{-/-}$ organoids.
(A) One-way ANOVA followed by Tukey's multiple comparisons test was conducted for Figure 2D and (B-C) Supplementary Figure S1C-D. Statistical significance is indicated as follows: ns, not significant; *p ≤ 0.05; **p ≤ 0.01; ***p ≤ 0.001; ****p ≤ 0.0001.

SUPPLEMENTAL FIGURE S4

A

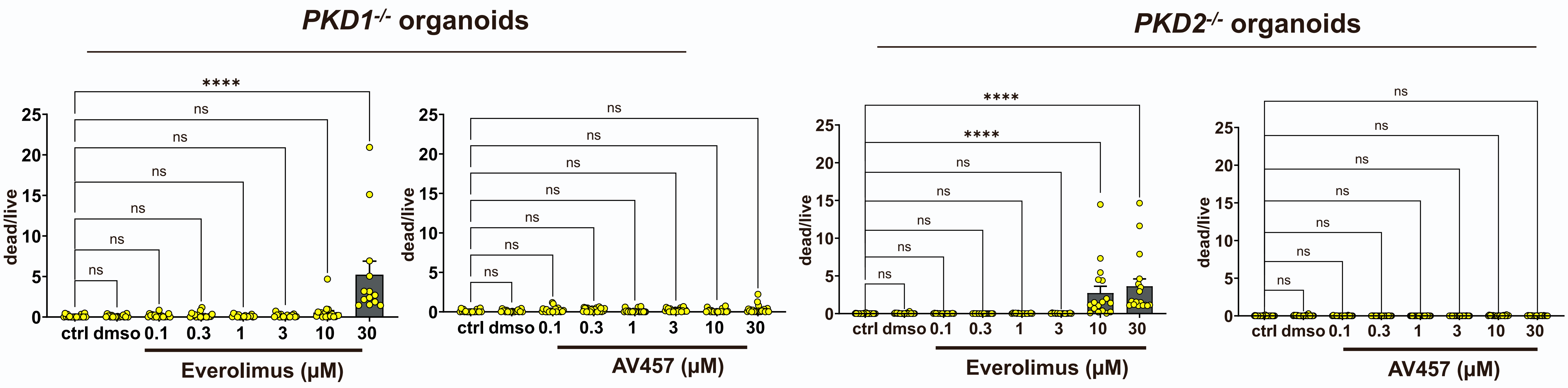

B

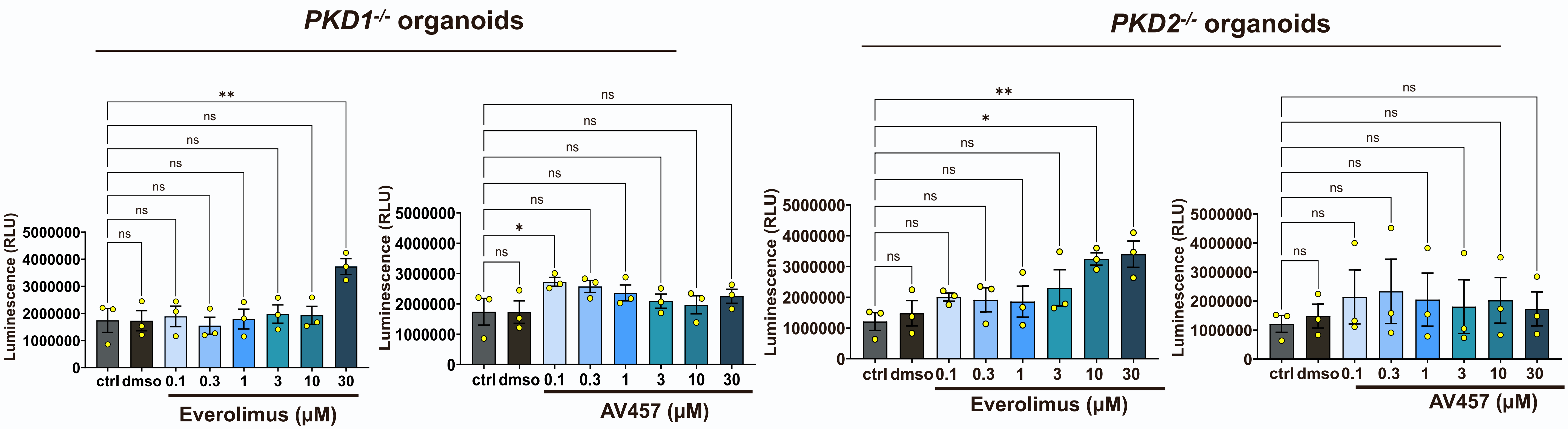

Supplemental Figure S4. AV457 shows reduced toxicity relative to everolimus in *PKD1*$^{-/-}$ and *PKD2*$^{-/-}$ cysts. (A) Live/Dead analysis using thresholding to measure the area (mean ± s.e.m.; n ≥ 20 organoids per condition pooled from 4 experiments with *PKD1*$^{-/-}$ or 3 experiments with *PKD2*$^{-/-}$ organoids). (B) LDH assays on day 7 of treatment (mean ± s.e.m.; n = 3 independent experiments). ns, not significant; *, p ≤ 0.05; **, p ≤ 0.01; ***, p ≤ 0.001; ****, p ≤ 0.0001.

SUPPLEMENTAL FIGURE S5

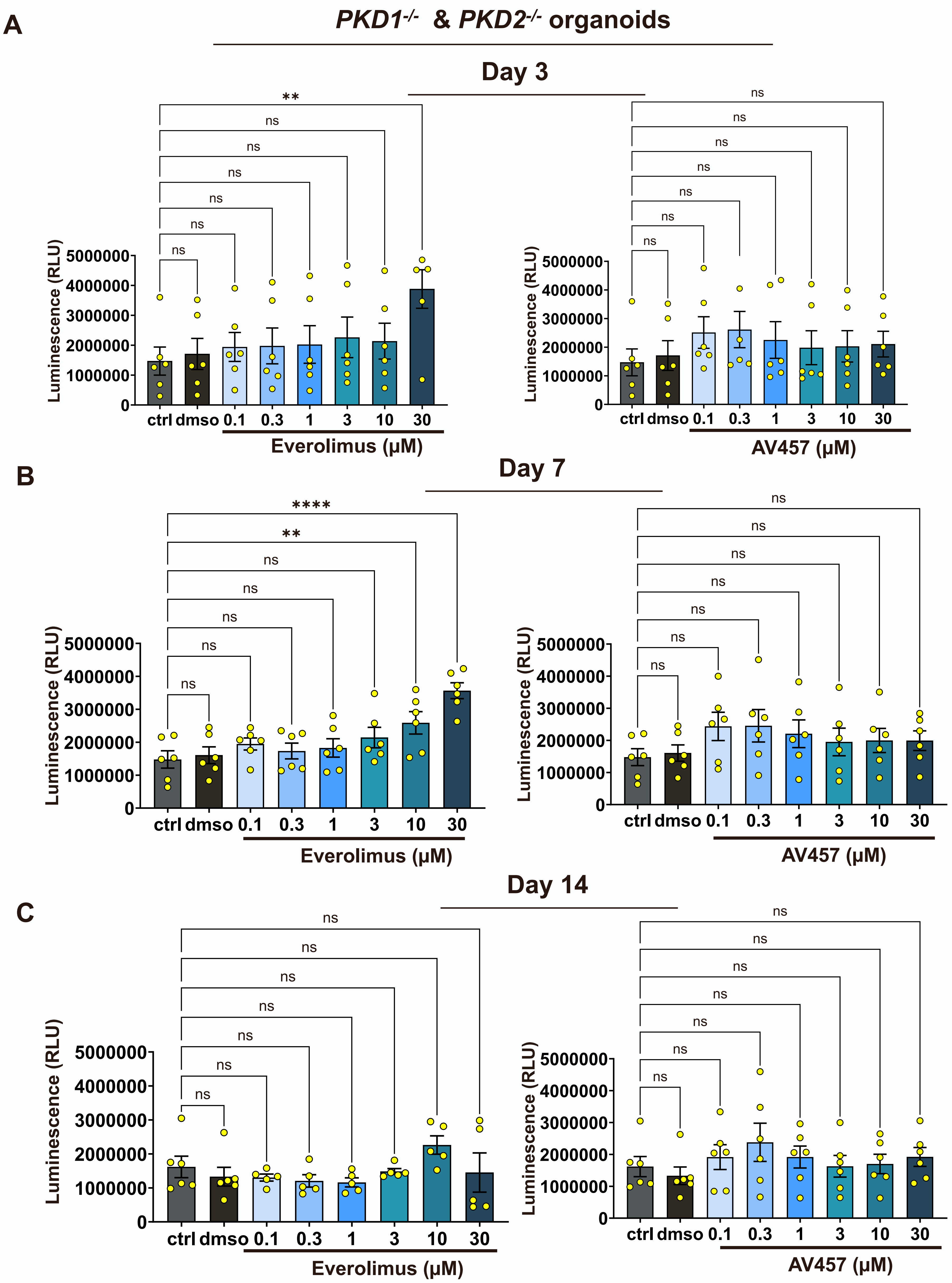

Supplemental Figure S5. LDH activity in PKD cysts is time sensitive. (A) LDH activity measured on day 3, (B) day 7, and (C) day 14 (mean ± s.e.m.; n = 6 independent experiments, three with *PKD1*$^{-/-}$ and three with *PKD2*$^{-/-}$ organoids; ns, not significant; *, $p \leq 0.05$; **, $p \leq 0.01$; ***, $p \leq 0.001$; ****, $p \leq 0.0001$).

SUPPLEMENTAL FIGURE S6

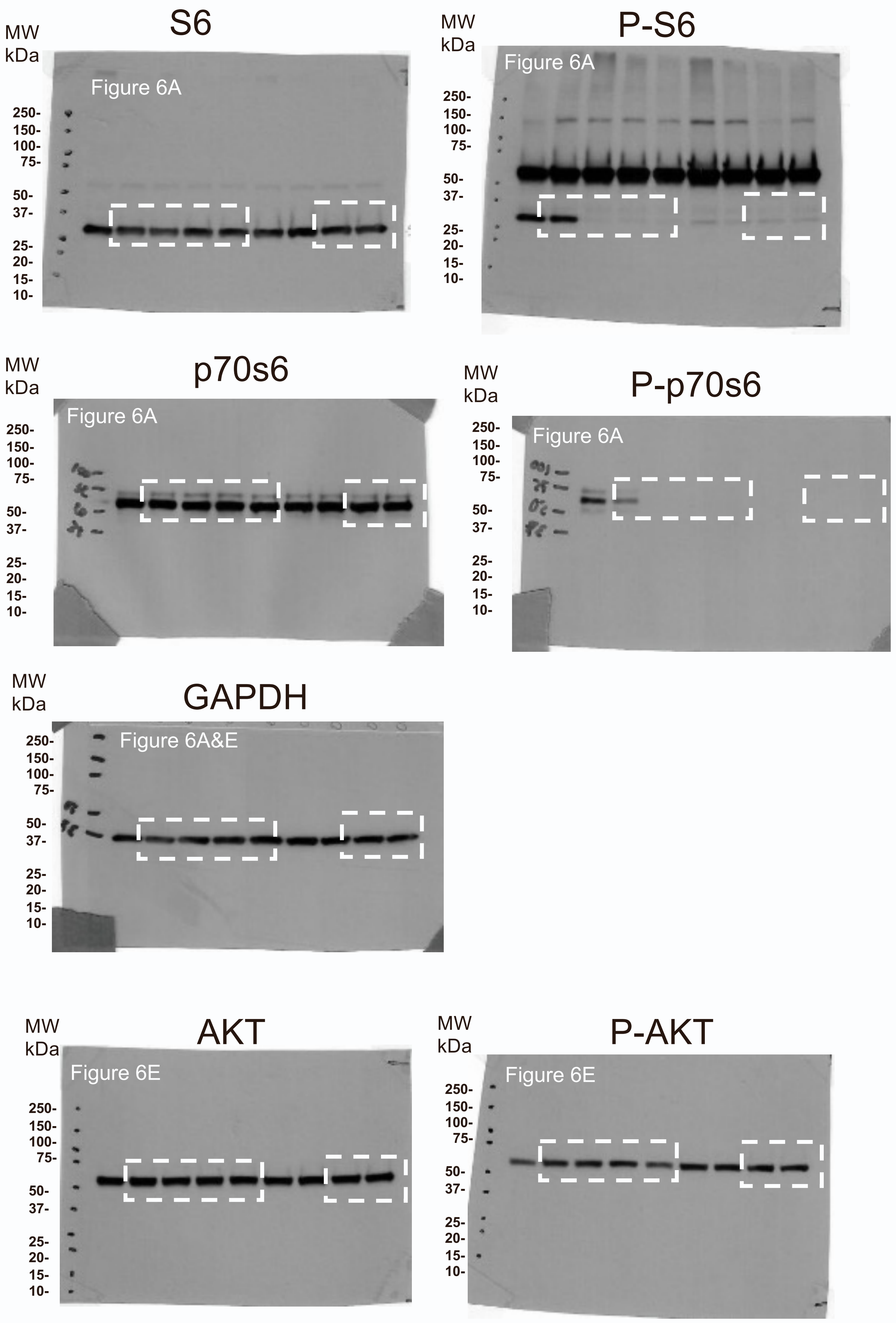

Supplementary Figure S6. mTOR inhibitors show target engagement in kidney organoids. Full representative Western blots from Figure 6. Cropped sections are boxed in a white outline.

SUPPLEMENTAL FIGURE S7

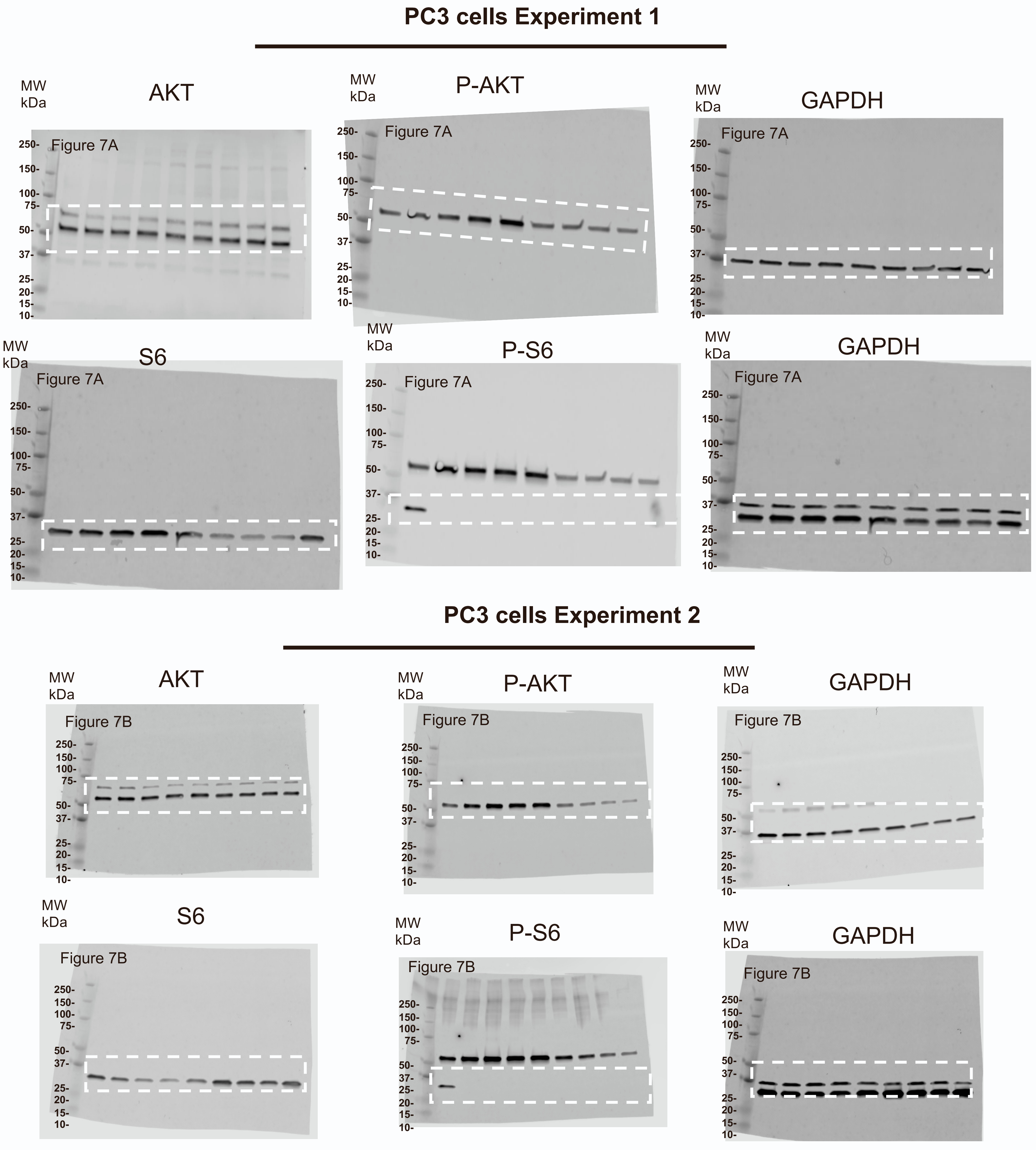

Supplementary Figure S7. **mTOR inhibitors show target engagement in PC3 cells.** Full representative Western blots from Figure 7. Cropped sections used in Figure 7 are outlined in white. For Experiment 1, the blots for S6, AKT, and their corresponding GAPDH initially had the control sample at the end of the blot. For consistency, these were cropped and repositioned with the control sample at the beginning, while the other blots remained unchanged.
